# Effect of Wood and Non-Wood Pulp Fiber Morphology on Laboratory-Simulated Tissue Paper Creping Performance: Functional Properties and Explainable AI

**DOI:** 10.1021/acsomega.6c08586

**Published:** 2026-10-06

**Authors:** Fernando Urdaneta, Ronald Marquez, Raine Viitala, Jorge Franco, Isabel Urdaneta, Daniel Saloni, Joel J. Pawlak, Hasan Jameel, Ronalds W. Gonzalez

**Affiliations:** † Sustainable and Alternative Fibers Initiative, Department of Forest Biomaterials, 6798 North Carolina State University, Raleigh, North Carolina 27695-8005, United States; ‡ LEPAMAP-PRODIS Research Group, 16738 University of Girona, C/Maria Aur′ elia Capmany, 61, 17003 Girona, Spain; § Department of Mechanical Engineering, School of Engineering, Aalto University, P.O. Box 14400, Aalto, FI 00076, Finland

## Abstract

Fiber morphology influences bonding, sheet structure, and the mechanical response of tissue webs during creping, but these relationships remain insufficiently quantified for high-yield nonwood pulps. In this work, alkaline peroxide mechanical pulps (APMP) from wheat straw, bamboo, miscanthus, switchgrass, and sorghum were blended with bleached eucalyptus kraft (BEK) and northern bleached softwood kraft (NBSK) pulps to produce 30 g/m^2^ laboratory handsheets. After simulating high-speed laboratory creping, their physical and mechanical properties were assessed. The results show that fiber morphology is a key factor: slender fibers (such as NBSK and bamboo APMP) yielded higher tensile strength, while less coarse fibers (such as BEK) enhanced softness. Nonwood APMP fibers consistently increased tissue bulk. The creping process boosted bulk by 80–150%, but also decreased tensile strength by 80–89%. An exploratory machine learning framework utilizing Random Forest regressors was developed to assess whether precursor sheet measurements could assist in predicting properties in this 12-furnish data set. Cross-validation and comparisons with linear regression indicated that the predictions were consistent with papermaking physics and softness forecasting. Since papermaking properties of APMP pulps, including softness, depend on multiple variables, such as the type of machine technology used to enhance softness, generalization remains difficult. Overall, strategic blending of 15–30% nonwood fibers provides a viable route to balance softness, strength, and bulk in tissue products, while feedstock and process-dependent standardized data sets are required before data-driven models can reliably assist furnish formulation.

## Graphical abstract

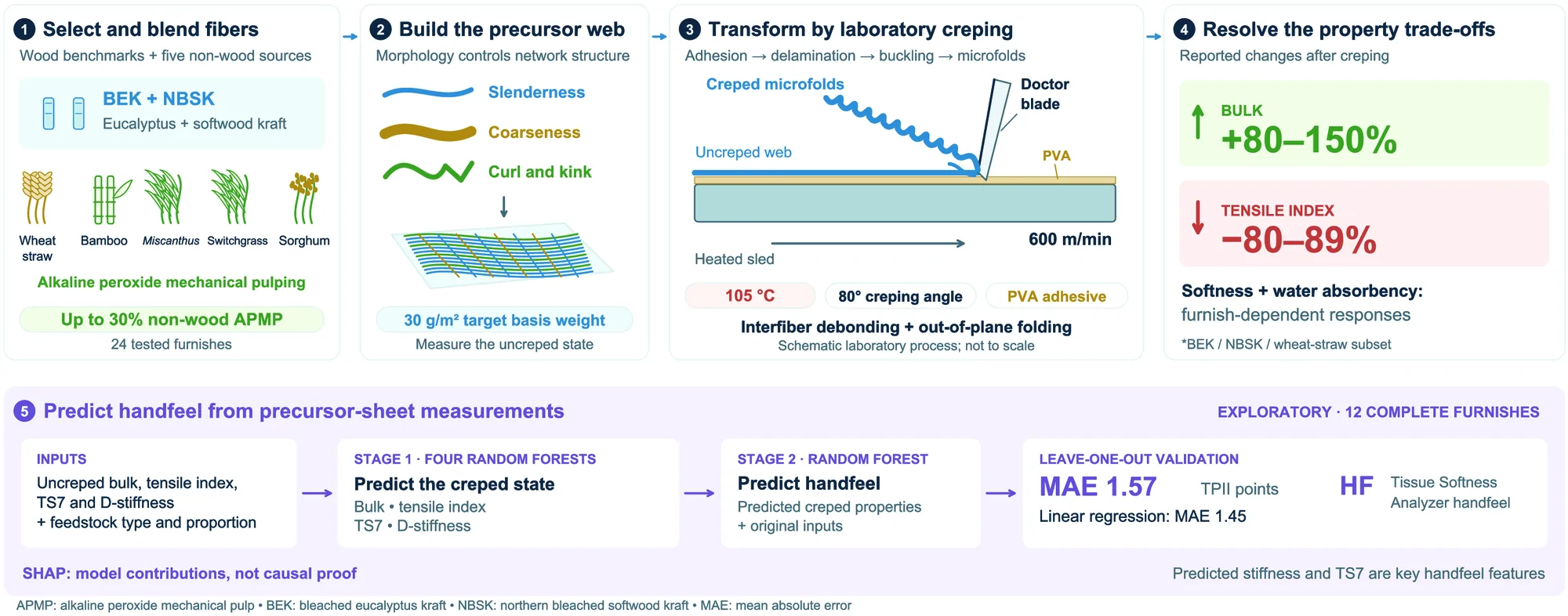

## 1 Introduction

The tissue paper industry has long relied on bleached eucalyptus kraft (BEK) and northern bleached softwood kraft (NBSK) fibers as primary raw materials. Their favorable fiber structure provides a good balance of softness, strength, bulk, and absorbency, enabling the production of high-performance bioproducts.^1,2^ However, increasing sustainability concerns and uncertainties about the supply of these traditional wood fibers present major challenges to industry stability and cost forecasts. Furthermore, market data indicate declining availability of high-quality recycled fiber (SOP), pressuring manufacturers to diversify fiber sources.^3^ The high silica content of nonwood fibers makes them difficult to process using kraft pulping. Alternatives like sulfite, chemo-thermal mechanical pulping, and chemi-mechanical pulping have been studied; this work will focus on alkaline peroxide mechanical pulping (APMP) based on previous results shown by Urdaneta et al.^7^ Nonwood fibers processed through APMP are being examined as cost-effective alternatives that broaden the fiber supply chain while supporting circular bioeconomy principles.^4,5^ Nonwood fibers are commercially viable; Packages Limited in Pakistan has operated the world’s first wheat straw chemithermomechanical pulping (CTMP) mill since 1991, producing 80 tons daily for corrugating and test-liner. This demonstrates opportunities and challenges, like high-shive content and low brightness, prompting ongoing research into alternatives like APMP to overcome these issues and target higher-value tissue products.^6^

APMP is a well-established high-yield pulping process that combines mechanical fiber liberation with simultaneous chemical bleaching using alkaline peroxide treatment.^7–9^ APMP was developed on hardwoods like eucalyptus and aspen, reaching over 80% ISO brightness.^10,11^ Later studies extended APMP to nonwood feedstocks: Kamyar et al. (2001) used APMP on bagasse with 10% NaOH and 3% hydrogen peroxide, yielding 76% and 77.7% ISO brightness, while Pan and Leary (2000a, 2000b) found wheat straw chromophores resist oxidation by hydrogen peroxide, limiting brightness to 50–55% ISO despite chelating and peroxide-stabilizing agents.^12–14^ This makes it especially suitable for processing nonwood raw materials, which often contain high silica levels that can create operational issues in kraft pulp production.^15^ Previous research by Urdaneta et al. (2024) demonstrated that APMP wheat straw pulps achieve high yields (around 75%), effectively retain fiber length, and produce tissue handsheets with greater bulk and water absorbency, while maintaining competitive softness compared to BEK benchmarks.^7–9^ The coarser fiber structure and higher lignin levels in wheat straw APMP pulp hinder fiber collapse and reduce interfiber bonding. This creates a more open, porous sheet that enhances bulk and water absorption, as discussed in previous work.^16^ Their follow-up study expanded this comparison to include APMP pulps from bamboo and miscanthus, showing that these nonwood fibers can enhance tensile strength and absorbency but often result in reduced softness. Strategic blending with wood kraft fibers, especially through partial substitution scenarios, has demonstrated potential to optimize the balance of strength, absorbency, and softness required for premium hygiene tissue products.^9^ The inherent variability and distinct morphological characteristics (length, coarseness, fines, stiffness) of these alternative fibers lead to complex and often nonintuitive effects on sheet properties.^9^

These trade-offs driven by fiber morphology are not exclusive to wheat straw APMP; they represent a wider trend seen across various nonwood pulping methods and research teams. Complementing these findings, the comprehensive assessment by de Assis et al. qualitatively and quantitatively characterized a range of wood (BEK, NBSK) and nonwood pulps (bamboo, wheat straw, others) for tissue manufacture.^2^ Their work explained how fiber morphological factors such as length, coarseness, fines content, and fiber population, critically influence tissue properties. Notably, nonwood pulps like bleached bamboo soda pulp can replace softwood fibers to provide strength without losing softness, while wheat straw soda pulps balance strength with intermediate absorbency and softness. They also noted that refining and mechanical treatment levels affect bulk, absorbency, and softness, highlighting the trade-offs in tissue production engineering. These insights support the rationale for using nonwood APMP fibers in tissue products while addressing challenges like fiber stiffness and sheet structure to meet product performance goals.

The creping process is an operation in tissue manufacturing that imparts the characteristic properties of softness, bulk, and absorbency. Creping is a crucial step in tissue manufacturing, especially in the light dry creping (LDC), the process can be described as a sequence of distinct physical events. First, a wet, continuous paper web is adhesively bonded to the surface of a large, heated, rotating cylinder (Yankee dryer).^1,2^ The sheet is rapidly dried on this polished surface. The transformation occurs when this dried, adhered sheet, traveling at high speeds (e.g., up to 30 m/s), is impacted by a stationary doctor blade. The process is a repeating cycle of three key events: interfacial delamination, compressive buckling, and microfold formation and collapse. This effectively generates interfiber debonding, resulting in a structure that is no longer a simple two-dimensional network but a complex three-dimensional one with a defined crepe wavelength and amplitude. The precise nature of this final structure is governed by a range of process parameters, including the creping angle (the angle between the blade and the tangent of the Yankee surface), the geometry of the blade, the properties of the adhesive coating on the Yankee, the machine speed, but also the type of feedstock/fiber morphology that is used to generate the paper sheet.^1,2^

The most significant changes involve a trade-off between strength and structural properties.^1,2,17^ The violent debonding and buckling action breaks a significant number of interfiber hydrogen bonds, leading to a reduction in the sheet’s tensile strength and stiffness.^2^ Concurrently, the formation of the microfold structure increases the sheet’s bulk, elongation, and water absorbency.^2,18^

Since fiber morphology, feedstock chemistry, and creping mechanics are interconnected in complex ways that are hard to analyze separately, a data-driven approach provides an alternative method to identify the key factors influencing creping performance. Existing literature establishes that the precise nature of the final creped structure is governed by process parameters such as creping angle, blade geometry, and machine speed, as well as the specific fiber morphology of the feedstock. Research indicates that fiber morphological traits, including length, coarseness, and stiffness critically influence how a sheet responds to these mechanical forces.^18–20^ This study uses machine learning to explore complex fiber-property relationships affecting creping performance. Limited sample size means it serves as an exploratory proof of concept, rather than providing a fully validated predictive model.^9^ Rather, it is benchmarked directly against simple linear regression to assess whether complex model structures offer any tangible advantage over traditional empirical relationships.

While Machine Learning (ML) often relies on large data sets, this research adopts a “small data” approach due to the limited number of pulp furnishes and blending ratios produced experimentally.^21,22^ Random Forest (RF) regressors were chosen for their robustness against overfitting and their ability to model nonlinear relationships in small samples through ensemble bagging. To keep the model transparent, SHapley Additive exPlanations (SHAP) were used. SHAP provides a mathematically grounded framework based on cooperative game theory to describe relative feature attributions within the trained model. Importantly, SHAP values reflect model-dependent statistical associations rather than direct physical causation, an essential distinction given the inherent collinearity among feedstock type, fiber morphology, and sheet structure. This interpretability serves as an exploratory check to evaluate whether the model’s learned patterns align with recognized papermaking mechanisms rather than reflecting arbitrary statistical artifacts of the small sample size.

This study investigates the effects of fiber morphology by including nonwood APMP fibers from wheat straw, bamboo, miscanthus, switchgrass, and sorghum in creped tissue paper made from a laboratory creping simulator. Conventional wood pulps (BEK and NBSK) are used as references.^9^ Key tissue properties, including caliper, bulk, tensile index, water absorbency, and softness (measured by EMTEC TSA handfeel factor), are examined to understand fiber-specific impacts and property trade-offs in creped tissue.

This work hypothesizes that the properties of the uncreped sheet influence its response during creping, which in turn determines its final creped features and perceived softness (TP2). Key properties include tensile strength, which affects resistance to buckling; initial bulk, which impacts potential *z*-direction expansion; and fiber morphology (stiffness, length, coarseness), which shapes microfold geometry. The study tests this by analyzing uncreped and creped tissue paper with 10–30% nonwood content, using a machine learning model to predict perceived softness and mechanical characteristics directly from uncreped-sheet data. The aim is to understand how fiber morphology and nonwood pulping affect creping results, providing insights into optimal fiber blends that balance softness, strength, and absorbency while supporting sustainable fiber substitution.

## 2 Methodology

### 2.1 Materials

#### 2.1.1 Fibers

Two commercial wood market pulps and five nonwood APMP pulps were used in this study. The wood pulps included BEK, and NBSK. Both market pulps were supplied in dry-lap form from commercial suppliers. The nonwood APMP pulps included wheat straw (WS), bamboo (BB), miscanthus (MS), switchgrass (SG), and sorghum (SH). Each feedstock was size-reduced through hammer-milling (0.5 in, 12.7 mm screen) and screened to remove fines and debris before being pulped using a laboratory-scale APMP process following the procedures described by Urdaneta et al.^7^ The resulting pulps were screened to remove shives using a 0.15 mm slotted screen, dewatered to approximately 30% consistency, and fluffed. Enough of each APMP pulp (minimum 500 g dry weight) was prepared to ensure consistent stock availability throughout the experimental program.

#### 2.1.2 Pulping Conditions

The APMP nonwood pulps used in this work were prepared using the conditions shown in Table 1.

**Table 1:** Operating Parameters for Alkaline Peroxide Mechanical Pulping (APMP) of Non-Wood Lignocellulosic Feedstocks. o.d. = oven-dry

Feedstock	NaOH charge (% o.d. fiber)	H_2_O_2_ charge (% o.d. fiber)	Liquor to fiber ratio	Temperature (°C)	Time (min)
Wheat straw	6.0	6.0	10:1	90	120
Bamboo	10.0	8.0	6:1	90	120
Miscanthus	8.0	6.0	10:1	90	120
Switchgrass	8.0	6.0	10:1	90	120
Sorghum	8.0	6.0	10:1	90	120

### 2.2 Furnish Preparation

Laboratory handsheets were prepared at a target basis weight of 30 g/m^2^ for all tests. Furnish designs included pure BEK, binary blends with NBSK or nonwood APMP pulps, and ternary blends containing BEK, NBSK, and nonwood APMP fibers. All stock blends were prepared at 0.3 % consistency in deionized water. BEK and all nonwood APMP pulps were used unrefined, while NBSK was refined in a PFI mill (TAPPI T 248 sp-00) to approximately 640 mL CSF. Table 2 lists all furnish compositions evaluated. These furnishes were selected based on two principles: first, substitution levels exceeding 30% will compromise properties beyond acceptable limits; second,^7^ production constraints at an industrial scale also play a role.^23^

**Table 2:** Furnish Compositions of Wood and Non-Wood Fiber Blends Evaluated for Creped Laboratory Handsheets^a^

Furnish Code	BEK (%)	NBSK (%)	WS (%)	BB (%)	MS (%)	SG (%)	SH (%)
100%BEK	100	0	0	0	0	0	0
BEK85-NBSK15	85	15	0	0	0	0	0
BEK55-NBSK45	55	45	0	0	0	0	0
BEK85-WS15	85	0	15	0	0	0	0
BEK85-NBSK5-WS10	85	5	10	0	0	0	0
BEK80-NBSK5-WS15	80	5	15	0	0	0	0
BEK75-NBSK10-WS15	75	10	15	0	0	0	0
BEK70-WS30	70	0	30	0	0	0	0
BEK55-NBSK15-WS30	55	15	30	0	0	0	0
BEK85-BB15	85	0	0	15	0	0	0
BEK85-NBSK5-BB10	85	5	0	10	0	0	0
BEK70-NBSK15-BB15	70	15	0	15	0	0	0
BEK70-BB30	70	0	0	30	0	0	0
BEK55-NBSK15-BB30	55	15	0	30	0	0	0
BEK85-MS15	85	0	0	0	15	0	0
BEK85-NBSK10-MS5	85	10	0	0	5	0	0
BEK70-NBSK15-MS15	70	15	0	0	15	0	0
BEK70-MS30	70	0	0	0	30	0	0
BEK55-NBSK15-MS30	55	15	0	0	30	0	0
BEK85-SG15	85	0	0	0	0	15	0
BEK70-NBSK15-SG15	70	15	0	0	0	15	0
BEK70-SG30	70	0	0	0	0	30	0
BEK70-NBSK15-SH15	70	15	0	0	0	0	15
BEK55-NBSK15-SH30	55	15	0	0	0	0	30

a BEK, bleached eucalyptus kraft; NBSK, northern bleached softwood kraft; WS, wheat straw; BB, bamboo; MS, miscanthus; SG, switchgrass; SH, sorghum. Values represent the percentage oven-dry fiber content in each furnish.

### 2.3 Handsheet Formation

Handsheets were formed in a British-style laboratory sheet former following TAPPI T 205 procedures.^24^ After formation, each sheet was couched using two blotter papers. The wet handsheets were then sandwiched between blotters, stacked, and sealed in a polyethylene bag for 24 h to maintain moisture before creping and to ensure uniform moisture distribution across the batch. The consistency of each batch was measured before creping and averaged approximately 60% consistency.

### 2.4 Laboratory-Scale Creping Simulation

Creping of the laboratory handsheets was performed using a high-speed creping simulator as described in Hämäläinen et al. Figure 1 shows the result of creping using the simulator.^18^ This device replicates the creping process parameters, including machine speed, adhesive application, and blade geometry. The simulator features a horizontally oriented, electrically heated sled that simulates the Yankee dryer surface. The sled’s temperature is programmable and controllable up to 125 °C; experiments in this study were conducted at 105 °C.

**Figure 1:**
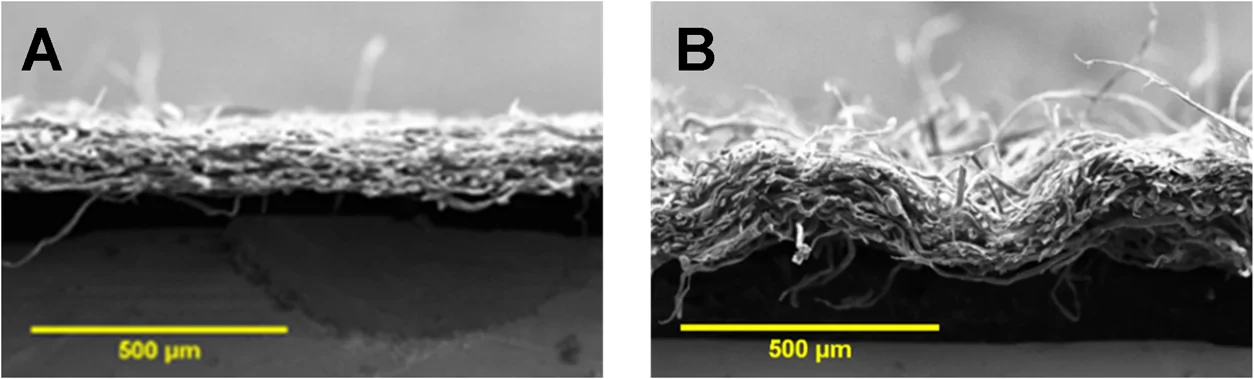
Surface morphology of tissue handsheets before and after creping. Scanning electron microscopy (SEM) images of a laboratory handsheet produced from the high-speed creping simulator. (A) Uncreped handsheet showing a planar fiber network; (B) creped handsheet showing the resulting microfold structure.

Creping trials began with each wet handsheet applied onto the press felt and transferred by a servo-driven press roll onto the sled. The simulator’s design enables accurate speed control between 0 and 600 m/min; for all experiments, the creping speed was set to 600 m/min. Prior to creping, a poly­(vinyl alcohol)–based (PVA) adhesive was sprayed onto the sled surface, using a pressurized feeding tank and nozzle, at a dosage of 1.0 mg/cm^2^. The sled, equipped with changeable surfaces, accelerates to the preset speed and traverses the coating and paper application zones before entering the creping station.

The creping blade is held at a controlled setup angle, delivering a creping angle of 80° for this study, which yields a final product with enhanced softness and microfolding. Standard ceramic-coated steel blades were used. Creping occurs as the sled transports the adhesive-bonded wet web to the creping blade, initiating sheet delamination, and micro- and macro-fold formation.

Each creped handsheet was collected postcreping and stored for subsequent property measurements. The simulator’s operation included cleaning of the sled surface between runs to maintain adhesive uniformity and avoid legacy effects from prior sheets. Before testing, the creped handsheets were stretched using the universal testing machine used for tensile strength measurements, the creped ratio obtained was on average 25.5% ± 4.4%.

### 2.5 Paper Properties Measurement of Creped Handsheets

Samples were conditioned at 23 °C and 50% relative humidity following TAPPI T 402 sp-13.^25^ Basis weight was measured according to TAPPI T 410 om-16,^26^ and grammage (g/m^2^) was calculated based on the area of the handsheet (0.002 m^2^). Caliper (thickness) and bulk were measured with a micrometer under 2 kPa pressure following ISO 12625–3:2014.^27^ The bulk was calculated as thickness divided by basis weight. Mechanical properties, including tensile strength and tensile index, were measured on a universal testing machine per TAPPI T 494 ^28^ using 50 mm wide test strips pulled at 14 mm/min with a test length of 100 mm. Water absorbency was measured following the ISO 12625–8:2016 basket-immersion method,^29^ where samples were immersed in water and water uptake was quantified gravimetrically. For softness evaluation, the Emtec Tissue Softness Analyzer (TSA) was employed to measure parameters such as TS7, which relates to fiber flexibility and surface traits, and TS750, which indicates surface smoothness. The algorithm used to calculate handfeel was TPII.

### 2.6 Statistical Analysis and Data Processing

Pearson correlation coefficients were calculated between all measured properties to identify significant relationships (*p* < 0.05). The correlation matrix and statistical tests were performed in Python (version 3.12.12) with the pandas (version 2.2.2), NumPy (version 2.0.2), and SciPy libraries (version 1.16.3). To assess differences among furnishes in tensile index, bulk, and HF_TPII, one-way ANOVA and Tukey’s HSD test were performed using means and SDs from Table 3. These tests were on 5 handsheets per furnish. Full results are in Table S3.

**Table 3:** Measured Physical Properties of Creped Handsheets from Wood and Non-Wood Fiber Furnishes^a^ ^,^ ^b^

Furnish Code	Caliper (μm)	Bulk (cm^3^/g)	Tensile index (N·m/g)	Water absorbency (g/g)	HandFeel (TPII)
100%BEK	379.0 ± 15.6	9.2 ± 0.40	1.0 ± 0.07	5.2 ± 0.26	88.2 ± 6.7
BEK85-NBSK15	322.0 ± 18.8	7.8 ± 0.42	1.2 ± 0.07	9.9 ± 0.90	86.8 ± 6.2
BEK55-NBSK45	291.8 ± 15.2	7.1 ± 0.26	2.1 ± 0.17	9.5 ± 0.81	79.5 ± 4.1
BEK85-WS15	371.0 ± 17.8	10.0 ± 0.45	1.3 ± 0.10	11.1 ± 0.96	84.5 ± 4.7
BEK85-NBSK5-WS10	414.0 ± 14.4	11.1 ± 0.53	1.2 ± 0.09	10.6 ± 0.94	86.4 ± 4.5
BEK80-NBSK5-WS15	414.0 ± 14.4	11.1 ± 0.35	1.5 ± 0.12	10.6 ± 0.57	82.5 ± 6.8
BEK75-NBSK10-WS15	362.6 ± 11.5	9.7 ± 0.47	1.6 ± 0.11	10.9 ± 0.74	80.5 ± 5.3
BEK70-WS30	344.0 ± 19.3	9.2 ± 0.32	1.7 ± 0.13	10.6 ± 0.59	81.1 ± 6.1
BEK55-NBSK15-WS30	347.9 ± 16.7	9.4 ± 0.30	2.6 ± 0.14	10.9 ± 1.02	78.5 ± 7.5
BEK85-BB15	345.8 ± 17.7	9.4 ± 0.55	1.3 ± 0.07	10.0 ± 0.81	89.2 ± 5.6
BEK85-NBSK5-BB10	356.0 ± 10.9	9.7 ± 0.57	0.9 ± 0.05	10.0 ± 0.67	87.1 ± 6.1
BEK70-NBSK15-BB15	320.0 ± 18.9	8.7 ± 0.47	1.8 ± 0.11	10.0 ± 0.53	79.8 ± 7.0
BEK70-BB30	331.8 ± 18.2	9.0 ± 0.35	1.8 ± 0.11	9.3 ± 0.61	84.3 ± 5.2
BEK55-NBSK15-BB30	346.6 ± 12.6	9.4 ± 0.31	2.0 ± 0.12	10.0 ± 0.66	79.0 ± 4.3
BEK85-MS15	415.5 ± 14.7	8.3 ± 0.42	0.8 ± 0.06	9.0 ± 0.78	90.7 ± 5.8
BEK85-NBSK10-MS5	371.8 ± 13.2	7.5 ± 0.32	1.2 ± 0.07	9.3 ± 0.76	89.4 ± 5.2
BEK70-NBSK15-MS15	318.0 ± 12.4	6.4 ± 0.22	1.3 ± 0.08	9.4 ± 0.89	84.5 ± 8.2
BEK70-MS30	383.0 ± 17.5	7.7 ± 0.35	0.6 ± 0.04	9.7 ± 0.71	86.4 ± 7.8
BEK55-NBSK15-MS30	374.0 ± 16.1	7.5 ± 0.23	1.7 ± 0.09	9.1 ± 0.51	82.9 ± 6.8
BEK85-SG15	465.0 ± 18.0	10.7 ± 0.61	1.0 ± 0.07	10.3 ± 0.88	87.8 ± 8.2
BEK70-NBSK15-SG15	378.0 ± 18.3	8.7 ± 0.33	0.9 ± 0.05	10.9 ± 0.96	80.5 ± 7.3
BEK70-SG30	382.0 ± 13.1	8.8 ± 0.44	0.9 ± 0.07	10.9 ± 0.85	86.4 ± 5.1
BEK70-NBSK15-SH15	270.0 ± 10.5	8.3 ± 0.33	3.0 ± 0.22	10.3 ± 0.91	72.7 ± 6.9
BEK55-NBSK15-SH30	267.1 ± 10.9	8.2 ± 0.37	3.1 ± 0.17	10.3 ± 0.77	58.7 ± 4.5

a TPII scale ranges from 0 (roughest) to 100 (softest).

b TPII, Total Handfeel index (Tissue Softness Analyzer scale).

### 2.7 Data Set Curation and Feature Engineering

The analysis was performed on a curated data set of 12 unique pulp furnishes, with complete physical property measurements available for both the uncreped precursor sheets and the final creped tissue sheets. This data set includes a range of conventional wood pulps (NBSK and BEK) and alternative nonwood feedstocks, such as Wheat straw, Miscanthus, Bamboo, and Sorghum. The input variables (features) were derived from the uncreped sheets and consisted of four physical properties: Bulk (cm^3^/g), Tensile Index (N·m/g), TS7 (a measure of fiber-level softness), and D_Stiffness (sheet stiffness). The target variables (outputs) included the corresponding properties of the creped sheets, as well as the final handfeel softness value, HF TPII, measured by a Tissue Softness Analyzer (TSA). To improve the model’s predictive capability, two separate feature engineering steps were applied to the raw data: Nonwood Percentage Extraction: A numerical feature, Non_Wood_Percent, was created by parsing the alphanumeric Furnish_Code using a regular expression. This parser identified nonwood pulp indicators (e.g., “WS”, “MS”, “BB”, “SG”) and extracted the associated percentages, providing the model with a quantitative measure of alternative fiber content. For conventional furnishes, this value was set to zero. Feedstock Type Encoding: The categorical feature “Nonwood”, which contains feedstock types such as “Wheat straw” and “Miscanthus”, was converted to a numeric format suitable for machine learning via one-hot encoding. This was achieved by employing pd.get_dummies from the pandas library, resulting in new columns prefixed with “Feedstock_” (e.g., “Feedstock_Wheat_straw”). This process generated a set of new binary features (e.g., Feedstock_Wheat_straw, Feedstock_Bamboo, Feedstock_non), where the value of “1” indicates the presence of that specific feedstock in the furnish and “0” indicates its absence. This enables the model to learn the unique, nonlinear influence of each fiber type on the final properties.

### 2.8 Chained Regression Modeling Framework

Given the small data set size and the complexity of the physical transformation during creping, an exploratory multistage chained regression framework was investigated. This approach decomposes the prediction task into two sequential stages that mimic the physical cause-and-effect pathway of the manufacturing process (Uncreped State → Creped State → Final Softness), thereby structuring the intermediate feature space to enhance physical interpretability. All models within this framework were instances of RandomForestRegressor from the scikit-learn library in Python, selected for their robustness with small data sets and ability to capture nonlinear interactions.

Random Forest is a robust predictive model made of diverse decision trees. It uses Bagging, where each tree is trained on a different sample of data, and the Random Subspace Method, which considers only a subset of features at each split. In regression, predictions are averaged, reducing variance and the risk of overfitting. The methodology utilized consisted of the following stages:

#### 2.8.1 Step 1: Intermediate Property Prediction

The first stage of the chained regression framework represents the mechanical transformation of the sheet induced by the creping process. Rather than directly predicting final softness, this intermediate step models how the uncreped sheet properties evolve after creping, thereby structuring the modeling pipeline in accordance with the physical manufacturing sequence with an exploratory reduction of prediction complexity in a data-constrained setting.

Four independent Random Forest regressors were trained to map the sheet’s precreping state to its corresponding creped physical properties. The input vector comprised the uncreped sheet descriptors (Bulk, Tensile_Index, TS7, and D_Stiffness), together with engineered feedstock features, including the percentage of nonwood fibers and one-hot encoded categorical feedstock variables. Each regressor was trained to predict a single creped property (Creped_Bulk, Creped_Tensile_Index, Creped_TS7, or Creped_D_Stiffness), resulting in a structured representation of the postcreping physical state.

By decomposing the transformation into property-specific regressors, the framework reflects the sequential transitions inherent to the creping process while improving model interpretability under small-sample conditions.

#### 2.8.2 Stage 2: Final Softness Prediction

The second stage of the framework leverages the intermediate predictions generated in Stage 1 to estimate the final handfeel softness of the creped sheet (HF_TPII). Rather than relying solely on the original uncreped descriptors, this stage incorporates the predicted creped physical state, thereby reflecting the full transformation pathway of the material.

The input space was constructed by concatenating the original precreping features with the predicted creped properties obtained from the four intermediate Random Forest models (e.g., Pred_Creped_Bulk, Pred_Creped_Tensile_Index, Pred_Creped_TS7, and Pred_Creped_D_Stiffness). This augmented representation enables the final regressor to operate on a physically enriched feature space that approximates the complete mechanical state of the sheet after creping.

A Random Forest regressor was then trained to predict the final softness metric (HF_TPII). By structuring the model in this chained manner, the framework preserves the physical sequence of the manufacturing process (Uncreped → Creped → Softness), improves transparency, and allows evaluation of intermediate physical descriptors in a small-sample regime.

#### 2.8.3 Model Validation and Interpretation

Due to the small data set size (*n* = 12), a conventional train-test split would yield an unreliable estimate of model performance. Therefore, Leave-One-Out Cross-Validation (LOOCV) was employed as a robust validation strategy for the final, chained model. In this procedure, the model is trained 12 times, each time using 11 samples for training and the single remaining sample for testing. This ensures that every sample is used as a test point exactly once, providing a rigorous and unbiased estimate of the model’s generalization performance on unseen data.

The performance metric for the cross-validation was Root Mean Squared Error (RMSE). This metric was chosen specifically because the Coefficient of Determination (*R* ^2^) is mathematically undefined for a test set of size one, as is the case in each fold of LOOCV.

Finally, to evaluate the internal decision logic and behavior of the trained Random Forest models in depth, SHAP (SHapley Additive exPlanations) analysis was conducted. This approach provides a consistent, mathematically grounded attribution score for each feature in every prediction. Crucially, SHAP values indicate the relative contribution of a feature to the model’s output and do not demonstrate physical causation. In this experimental data set, feedstock type, fiber morphology, and handsheet properties exhibit substantial collinearity; consequently, SHAP attributions distribute importance across correlated features rather than establishing causal mechanisms. We therefore interpret SHAP results strictly as descriptive diagnostic patterns that examine whether model behaviors align with known papermaking principles. This instance level interpretability provides transparency regarding the model’s internal weighting, which is valuable for assessing the consistency of data-driven associations in creped tissue systems.

## 3 Results and Discussion

### 3.1 Fiber Morphology

The fiber morphology was determined optically using a fiber quality analyzer, and the results are shown in Figure 2.

**Figure 2:**
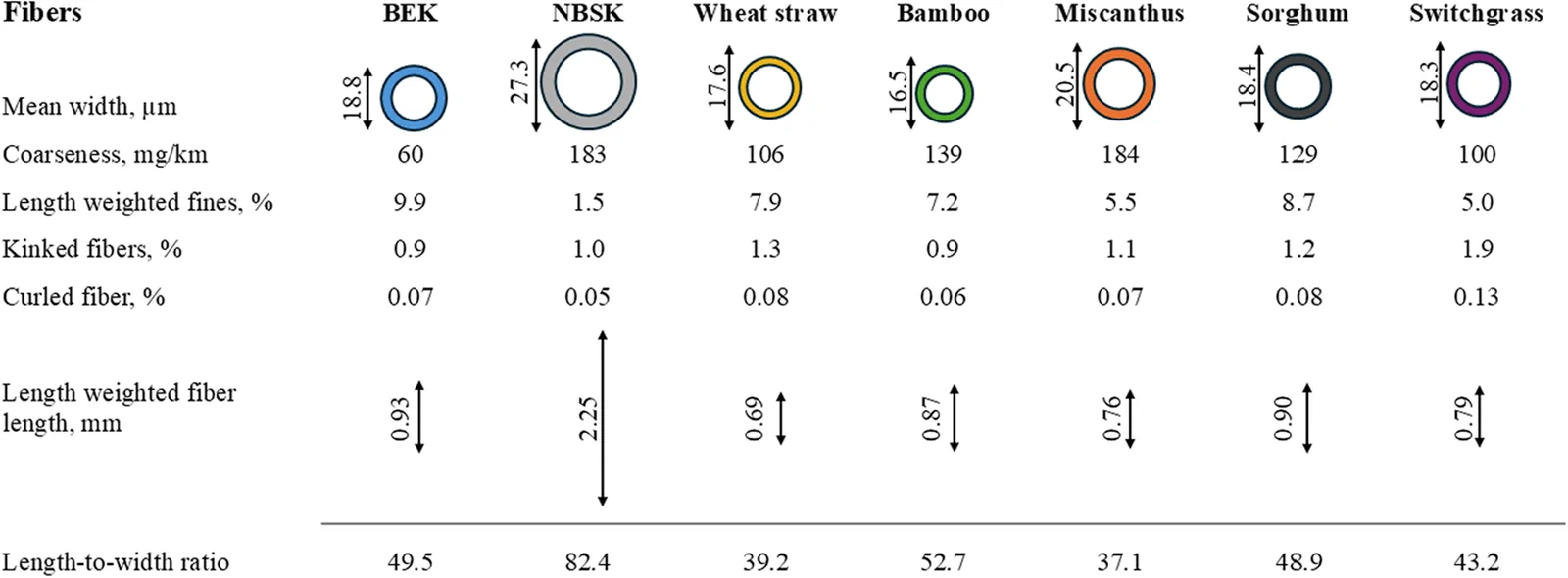
Fiber morphology in wood and nonwood pulps. A comparison of features such as length, width, coarseness, and fines content for bleached eucalyptus kraft (BEK) and northern bleached softwood kraft (NBSK) pulps versus nonwood alkaline peroxide mechanical pulps (APMP) from wheat straw (WS), bamboo (BB), miscanthus (MS), switchgrass (SG), and sorghum (SH). Values are averages obtained through optical fiber quality analysis.

NBSK was the longest wood fiber and the longest overall. All nonwood fibers were shorter than BEK; fiber length correlates with tensile strength, and thus shorter fibers can be expected to produce weaker but softer paper.^7^ The fines content of the nonwood fibers fell within two groups: The first group, which included wheat straw, bamboo, and sorghum, had a higher fine content of around ∼7.5%, while the second group, which included miscanthus and switchgrass, had a lower fine content of around ∼5.5%. It is important to note that the nonwood pulps were washed and screened, which may have reduced their fines content. Regarding wood fibers, BEK had the highest fines content of all pulps used in this study, while NBSK had the lowest. Fines can be divided into fibril-like fines and flour-like fines.^30–32^ The former can form a bond between fibers, while the latter generally do not. This means that fibril fines can improve the tensile properties, decrease bulk, and overall hurt softness. On the other hand, flour-like fines interrupt the formation of bonds, having the opposite effect on tensile properties and bulk.^30,33,34^ Because nonwood fines are known to differ fundamentally from wood-derived fibrillar and flake-like fines, often consisting of largely intact parenchyma and epidermal cell fragments rather than broken fiber-wall material, and because standard fractionation methods such as Bauer-McNett and optical analysis are known to under or overestimate their true content in nonwood pulps, direct morphological characterization of the fines fraction for these specific APMP pulps remains an important priority for future work.^31,32,35,36^

Curliness and kink account for deformation in the fiber; curliness refers to curvatures along the fiber length. In contrast, kink corresponds to abrupt changes in the curvature of the fibers (sharp bends). These deformations affect how the fibers pack together; the curlier the fibers and the more kinks they have, the greater the increase in water absorbency, bulk, and softness, while decreasing tensile properties^35,37,38^ The results show that APMP switchgrass yielded the most deformed fibers overall for this study.

Both width and coarseness are generally correlated; a less coarse fiber has a higher tendency to collapse and thus provides a higher surface for bonds, producing a stronger paper, while a coarser fiber creates a more open and porous network, promoting bulk and water absorbency.^7–9^ At a constant mass, paper produced with a lower coarse fiber content will have more fibers, leading to a higher number of fiber ends on the surface, creating a velvety feel and increasing surface softness. On the other hand, a coarser fiber will produce a stiffer paper that feels less soft.^7–9^

Similar to fiber length, the slenderness ratio correlates well with tensile properties; a slender fiber has higher bonding and entanglement potential, although an excessively high ratio can produce rough paper. Thus, there is a trade-off between slenderness and softness that needs to be considered. A ratio between 50 and 80 is considered ideal for papermaking. Based on this criterion, BEK, NBSK, APMP bamboo, and sorghum are within the optimal range, while the rest have a lower than optimal slenderness ratio.^2,35^

The results shown in Figure 2 are average values; in reality, pulp is a complex mixture of fibers with different morphological characteristics. The distribution of length and width is shown in Figure 3. BEK differs from the rest of the pulp due to its narrow length and width distribution, indicating the homogeneity of the raw material. Eucalyptus is usually grown in industrial plantations where species and ages are controlled to obtain pulp of reliable quality.^39,40^ On the other hand, the wide distribution of NBSK is a consequence of its heterogeneous source, in which different softwood species and ages are mixed.^2,41^ Overall, the APMP nonwood pulps showed a wide distribution in both figures; this is due to the heterogeneity of the raw material and the nature of mechanical pulping, in which fibers are pulled apart from the lignocellulosic matrix.^8,35,42,43^

**Figure 3:**
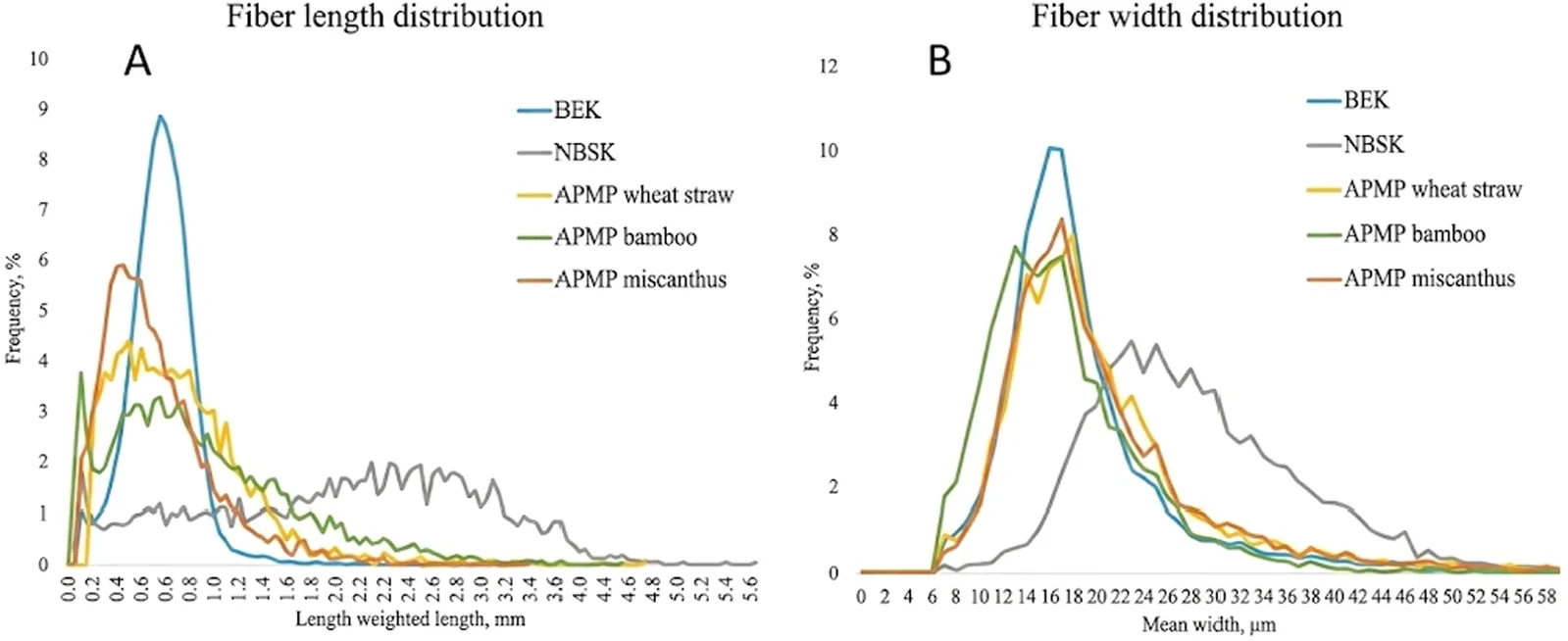
(A) Fiber length and (B) width distributions are shown through plots for BEK, NBSK, and nonwood APMP pulps (WS, BB, MS, SG, SH), based on optical fiber quality analysis. Reprinted with permission from ref.^6^ Copyright 2025 Springer.

The relation of coarseness and slenderness ratio is shown in Figure 4. This relationship can provide insight into the fiber’s performance. In the graph, we see a diagonal line from BEK to NBSK, indicating a positive correlation between coarseness and the slenderness ratio, at least for bleached chemical wood fibers. In the case of the nonwood APMP fiber, no clear correlation is observed; for example, APMP miscanthus was the coarsest fiber, yet it had the lowest slenderness ratio.

**Figure 4:**
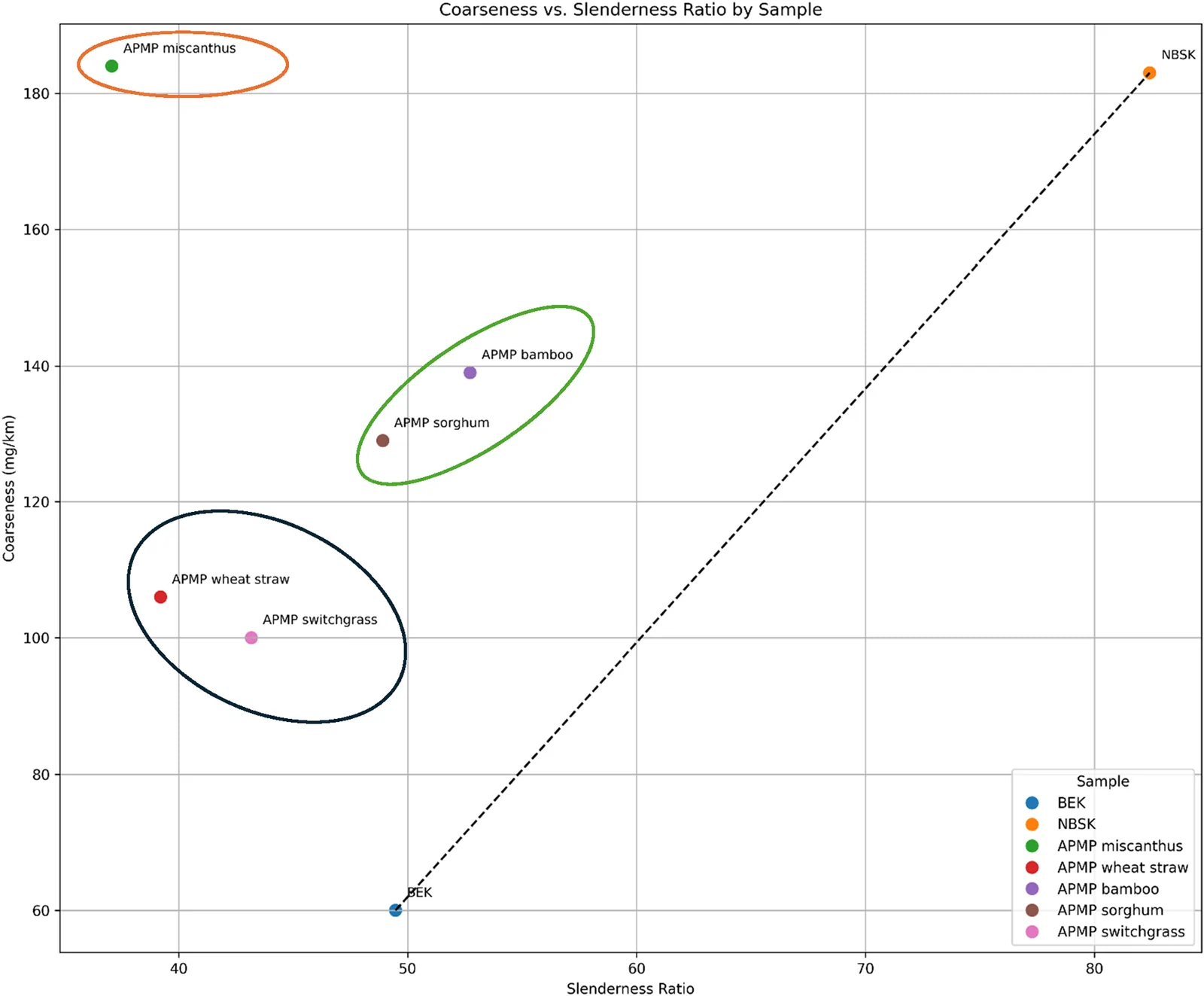
Coarseness-versus-slenderness ratio of wood and nonwood fibers. Scatter plot relating fiber coarseness (mg/m) to slenderness ratio (length/width) for BEK, NBSK, and nonwood APMP pulps (WS, BB, MS, SG, SH).

For the APMP nonwood fiber, three groups can be considered. The first group, composed of wheat straw and switchgrass, is coarser and has a lower slenderness ratio than BEK; this could indicate that these fibers will produce a bulkier and weaker paper than BEK. The second group of bamboo and sorghum sits midway between BEK and NBSK on the graph, indicating that these fibers will provide intermediate properties for paper. The final group made by miscanthus had the highest coarseness and the lowest slenderness ratio, meaning that it will form a bulky and weak paper.^35,44^

### 3.2 Effect of Morphology on Uncreped Handsheet Properties

Surface chemistry and fiber morphology influence the properties of uncreped handsheets, especially when comparing high-yield nonwood fibers to traditional wood pulps. Alternative fibers such as wheat straw and bamboo, processed through APMP, offer several benefits. Wheat straw fibers are longer (0.95–1.03 mm) than BEK pulp fibers (0.81 mm), which enhances fiber contact and tensile strength. Their inherent stiffness helps maintain a three-dimensional shape, preserving bulk and porosity, and improving water absorption. However, this same stiffness and coarseness- measured at 111 mg/m for miscanthus and 103 mg/m for bamboo compared to BEK’s 72 mg/m- can decrease softness, increase surface roughness (TS750), and fiber stiffness (TS7).^8^

Chemically, XPS shows APMP nonwood fibers have lower O/C ratios (51–53) than kraft pulps (0.63–75), indicating higher surface lignin. Though lignin hinders hydrogen bonding, APMP modifies the surface via carboxylation and oxidation, increasing swelling and bonding. This allows high-yield fibers to reach high tensile strength without extensive lignin removal. The balance between fiber length, surface chemistry, and bonding enables nonwood fibers to replace long-fiber softwood kraft, maintaining or improving bulk and absorbency.

### 3.3 Properties of Creped Tissue

The properties of the creped handsheets are presented in Table 3. A one-way ANOVA demonstrated that the furnish composition significantly influenced the tensile index, bulk, and HF_TPII (all *p* < 0.001; see Table S3). The first sample shown is made only from wood fibers, and the trend in properties is consistent with the data shown in Figure 4. As the NBSK content increased, the tensile index increased, while caliper, bulk, and softness (handfeel) decreased. As explained before, NBSK is a slender fiber, so by adding to the furnish, a stronger and more compact fiber network was formed, which also explains the decrease in both bulk and surface softness.^9,17,44^

For wheat straw, a clear trend consistent with the morphological data was not observed; wheat straw stands left and up from BEK in Figure 4; meaning that it was expected that including wheat straw would produce a bulkier yet weaker paper. However, when 15% of APMP wheat straw was used, the tensile index increased compared to 100% BEK; a similar effect was seen with the addition of 30% APMP wheat straw. On the other hand, when NBSK is present in the mix, the caliper of the handsheet increases.

For the case of bamboo, a trend more consistent with its morphology was observed. The higher coarseness of bamboo compared to BEK explains the increase in bulk compared to the 100% BEK handsheet; this increase in bulk also leads to an improvement in water absorbency. The greater coarseness of bamboo creates a thicker network with more voids, making the paper bulkier and providing more space to store water. The slenderness of the bamboo fiber was intermediate between BEK and NBSK, resulting in a higher bonding capacity than BEK and improving the tensile index of the handsheet, which increased as the bamboo content rose. The higher coarseness and bonding capacity reduce softness compared to the 100% BEK handsheet. BEK85-BB15 showed a higher HF_TPII than other bamboo furnishes, but this was not statistically significant (Tukey HSD, *p* > 0.05; Table S3) and should be seen as a trend rather than proof of an optimal bamboo level.

For miscanthus, the influence of its morphology can be seen in the BEK85-MS15 sample, where high bulk and softness were achieved, but it had a low tensile index. This is due to its unusually high coarseness and low slenderness ratio, which created a weak, bulky web that contributed to the perceived softness. It was observed that adding long fibers (NBSK) to the mixture reduced bulk and softness while increasing the tensile index, as expected. Additionally, at higher miscanthus concentrations (BEK70-MS30), the impact of its morphology is more evident, as it results in low tensile strength; however, the increased stiffness of miscanthus fibers decreases softness perception, countering the typical trade-off between tensile strength and softness.^2^ This can be explained by its unique combination of high coarseness and low slenderness ratio, highlighting the need for careful optimization when using this feedstock.

Switchgrass’s moderate slenderness ratio and coarseness produced intermediate properties. The BEK85-SG15 sample had higher bulk than the 100% BEK sample, but the introduction of stiffer fibers led to a decrease in softness. When NBSK was introduced, a densification of the sheet was observed as bulk decreased. This added to the higher roughness expected from more slender fibers, explaining the decrease in softness.^2^ The effect of slenderness is further observed in the mix BEK70-SG30, where NBSK is not present, and this resulted in a softer handsheet, even though bulk and tensile strength remained similar.

Lastly, sorghum showed the poorest performance in terms of softness. However, this could be due to the inclusion of long fibers (NBSK), as no sample containing only BEK and sorghum was evaluated. This softness deficit was statistically significant compared to nearly all other furnishes (Tukey HSD, *p* < 0.05 for sorghum-containing vs most others; Table S3), confirming sorghum’s poor softness is a robust effect, not just a trend. The combination of moderate coarseness and a slenderness ratio added to NBSK’s morphology created a stronger but rougher paper.

The correlation of different paper properties is shown in Figure S1. Caliper had a moderately high positive correlation with handfeel, consistent with previous studies that found a thicker sheet provides cushioning, making the paper feel softer. Surprisingly, bulk had a low positive correlation with softness. As expected, caliper and bulk had a negative correlation with the tensile index, a less bonded network results in a thicker sheet. The typical trade-off between tensile strength and softness was observed, as reflected in a strong negative correlation. Water absorbency showed a moderate positive correlation with bulk. The negative relationship between softness and water absorbency may be due to the use of mechanical pulp fibers. These fibers tend to be coarse, creating more voids where water can be stored.^2^ However, they are also stiffer, so as more nonwood mechanical fibers are added to the mix, a rougher and stiffer surface is created, lowering softness.^45^

### 3.4 Change in Properties after Creping

To ascertain the effect of creping in paper properties, creped and uncreped handhseet containing BEK, NBSK, and wheat straw were compared; results are shown in Figures 5 and 6.

**Figure 5:**
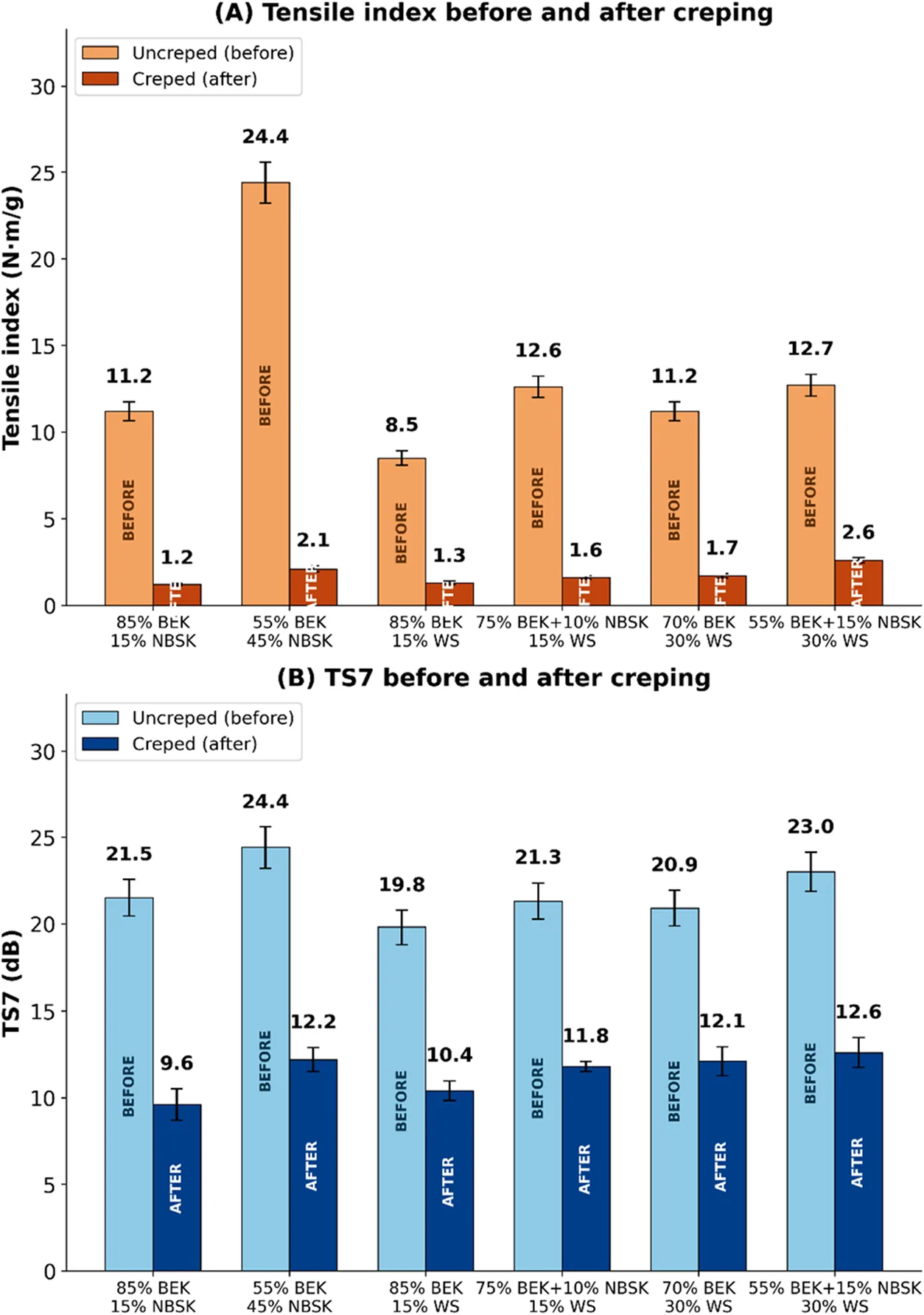
(A) Tensile strength and (B) softness changes after creping. Tensile index and handfeel (HF, TPII) values before and after creping for selected furnishes containing BEK, NBSK, and wheat straw (WS) at varying substitution levels.

**Figure 6:**
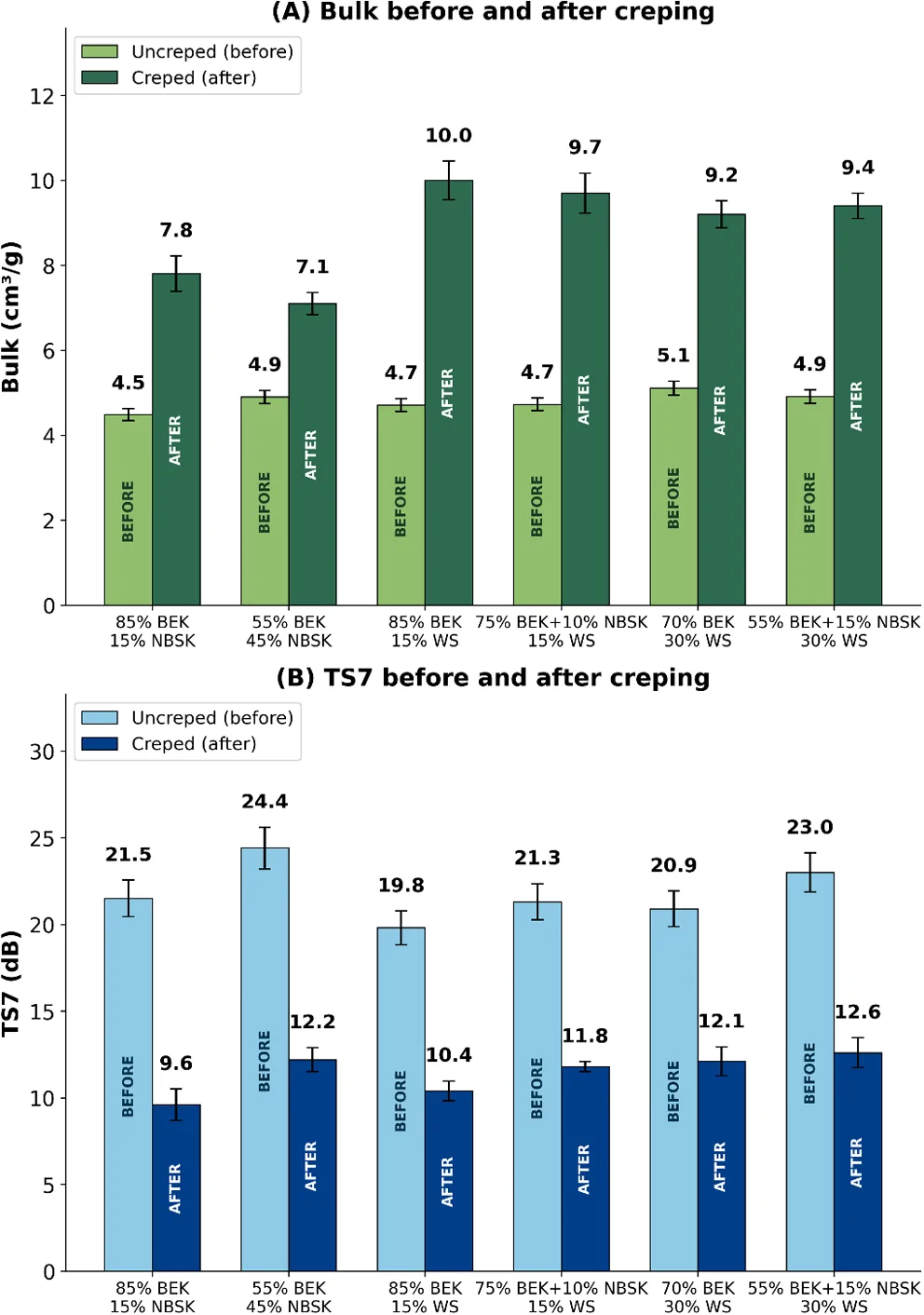
(A) Bulk and (B) softness changes after creping. Bulk (cm^3^/g) and handfeel (HF, TPII) values before and after creping for selected furnishes containing BEK, NBSK, and wheat straw (WS). Predicting creped tissue properties.

Tensile index loss (Figure 5) was between 80 to 89%. These results are higher than those reported by de Assis et al. in their study (∼68%) using the same machine.^2^ This could be due to differences in the fibers employed. De Assis et al. used chemical fibers, both virgin and recycled, and the only nonwood fiber used was bamboo; as shown in Figure 4 bamboo has higher slenderness than wheat straw, making it more likely to retain tensile index after creping, plus in de Assis’s study the bamboo fiber was obtained through soda pulping. With a slenderness ratio of 111, it is reasonable to expect better performance in tensile strength. The graph below (Figure 5) shows an expected relationship between tensile strength and softness; as tensile strength decreased, the paper became softer. The effect of wheat straw on paper properties was varied but could be modified by changing the proportions of BEK and NBSK. When all the NBSK was replaced by APMP wheat straw (85% BEK + 15% WS), a sharp decline in tensile strength was observed, but softness was also improved. The drop in tensile was reversed when either more wheat straw was added at the cost of removing BEK, or when NBSK was present in the furnish. In both cases, the tensile strength was similar to 85% BEK + 15% NBSK, but the softness decreased when BEK was removed.

As expected, creping increased the sheet’s bulk, from 80 to 150% (Figure 6). These results are more varied than those reported by de Assis et al.,^20^ who noted an increase of 100% to 110%. This could be because the fibers used in this study exhibited broader differences in morphology. Additionally, in this study, the creping speed was set at 10 m/s, whereas in the previous work, it was set at 2.5 m/s; higher speeds can result in thicker sheets.^18^ Finally, de Assis et al. used refined pulps, whereas in this study, only NBSK was lightly refined (1000 revolutions on a PFI mill). Refined pulps produce denser sheets.

#### 3.4.1 Overall Model Performance

We evaluated the performance of the chained architecture by comparing it with a single-stage Random Forest directly predicting HF_TPII from uncreped descriptors (Figure 7), as well as a standard linear regression baseline, using the LOOCV protocol (Table S2). The chained Random Forest model achieved an MAE of 1.570, outperforming the single-stage Random Forest (MAE = 2.182). However, it did not outperform the simple linear regression baseline, which achieved an MAE of 1.451. This result demonstrates that, for this 12-sample data set, the added architectural complexity of the nonlinear chained Random Forest is not justified by superior predictive accuracy. We retain the Random Forest model as an exploratory diagnostic tool for feature attribution via SHAP and to probe potential nonlinear trends, which may be useful at highly nonlinear industry settings. Residual and SHAP analyses are provided in the Supporting Information (Figures S14 and S15). On average, model predictions deviate from measured TSA values by approximately 1.57 handfeel points. However, as noted above, this level of accuracy is comparable to a simple linear baseline and must be viewed within the confines of an exploratory, small-sample pilot study.

**Figure 7:**
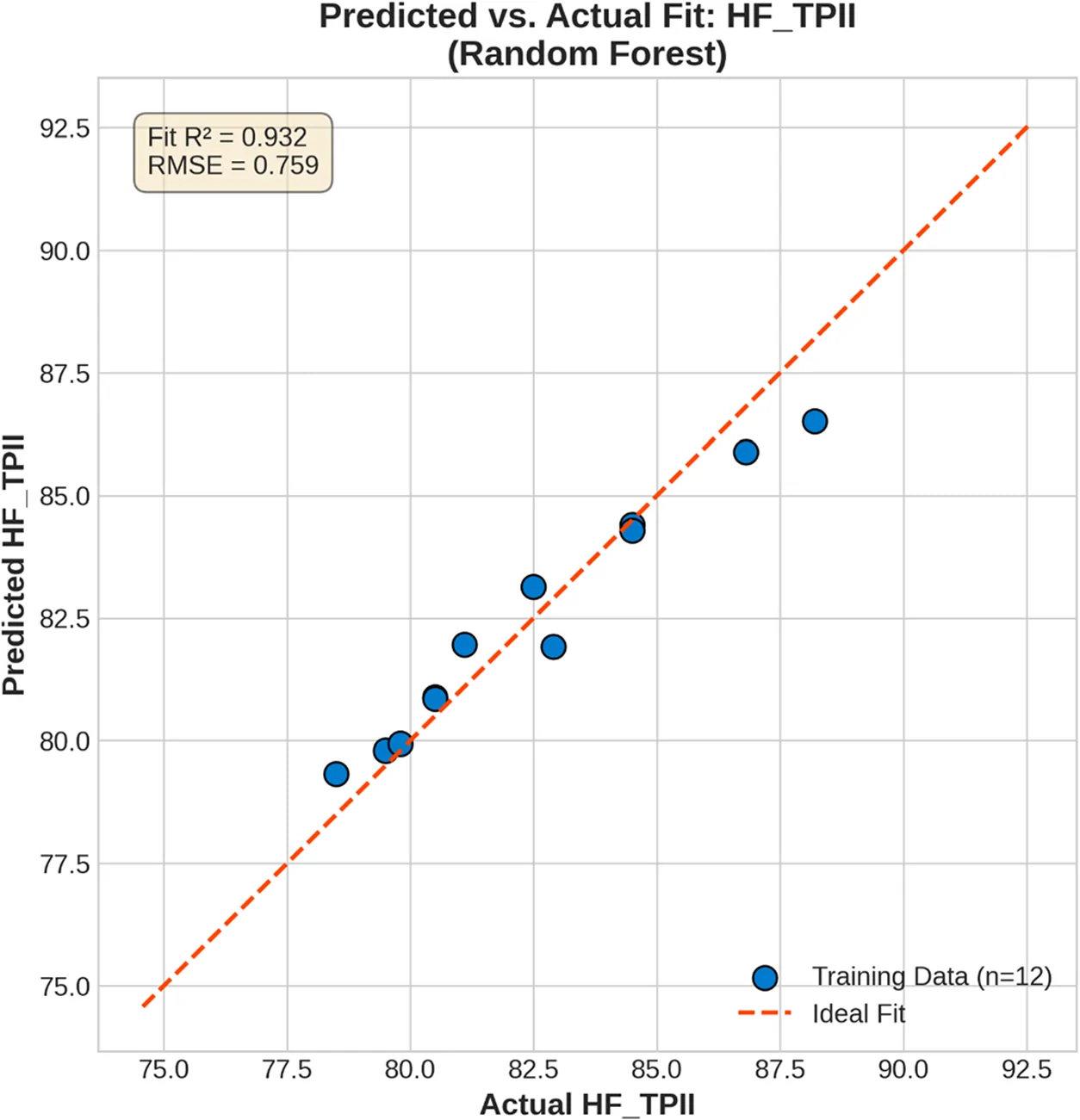
Chained Random Forest model performance for softness prediction. Predicted versus measured handfeel (HF, TPII) for creped handsheets (*n* = 12), generated using Leave-One-Out Cross-Validation (LOOCV) of the chained Random Forest regression model.

The individual RMSE scores for each of the 12 folds in the cross-validation were [3.497, 2.349, 1.237, 2.153, 0.890, 0.840, 0.260, 2.205, 1.517, 0.268, 2.786, 0.841]. The two folds with the lowest error rates (RMSE = 0.260, 0.268) are linked to furnishes with typical wood-dominant morphology. In contrast, the highest-error folds (RMSE = 3.497, 2.786) involve nonwood-rich furnishes that display unusual combinations of coarseness and slenderness. This pattern aligns with the model’s limited ability to generalize to less-represented areas of morphological space, especially when trained on only 11 points. The standard deviation of these errors was 0.983, resulting in a 95% confidence interval of ±1.966 around the mean. The range of errors suggests that while the model was moderately accurate for most samples (with errors below 1.0), its performance is less consistent for a few specific furnishes, which represent more challenging or unique transformations that the model struggles to predict when that specific point is excluded from the training set.

Analyzing the LOOCV results by furnish (Table S1) shows that the largest prediction errors were for BEK100 (+3.50), BEK85-NBSK15 (+2.35), and BEK55-NBSK15-MS30 (+2.79), while the smallest errors were for BEK85-WS15 (+0.26) and BEK70-NBSK15-BB15 (−0.27). Interestingly, the largest errors were not limited to nonwood-rich furnishes; the pure wood-fiber (BEK100) and a wood-dominant blend (BEK85-NBSK15) also had substantial errors. This pattern aligns with a known limitation of LOOCV on small, heterogeneous data sets: when a furnish resides at the extremes or in sparsely represented areas of the feature space, like the only 100% BEK sample or a high-miscanthus blend, removing it reduces local coverage in the remaining 11 training points. This diminishes the model’s ability to extrapolate accurately, leading to higher variability between folds (SD = 0.983; 95% CI = ±1.966 around the mean). This pattern does not suggest that predicting nonwood feedstocks is inherently more difficult than wood-based furnishes.

#### 3.4.2 Analysis of Intermediate and Final Models

The chained regression framework facilitated the analysis of both the intermediate physical property predictions and the final softness prediction, as illustrated in Figure 8. For the intermediate bulk model, results such as predicted versus actual creped bulk, residual distribution, and SHAP feature importance are shown in Figures S2, S3, and S4, respectively. Regarding tensile index prediction, the model performance and residuals are displayed in Figures S5 and S6, with the corresponding SHAP analysis in Figure 8 (main text) and Figure S7. Similar diagnostics for the TS7 softness metric are provided in Figures S8–S10, and for D-stiffness in Figures S11–S13. The in-sample Fit *R* ^2^ values, indicating how well the models explain the data, are also presented. The variance within the 12-sample data set on which they were trained was exceptionally high.

**Figure 8:**
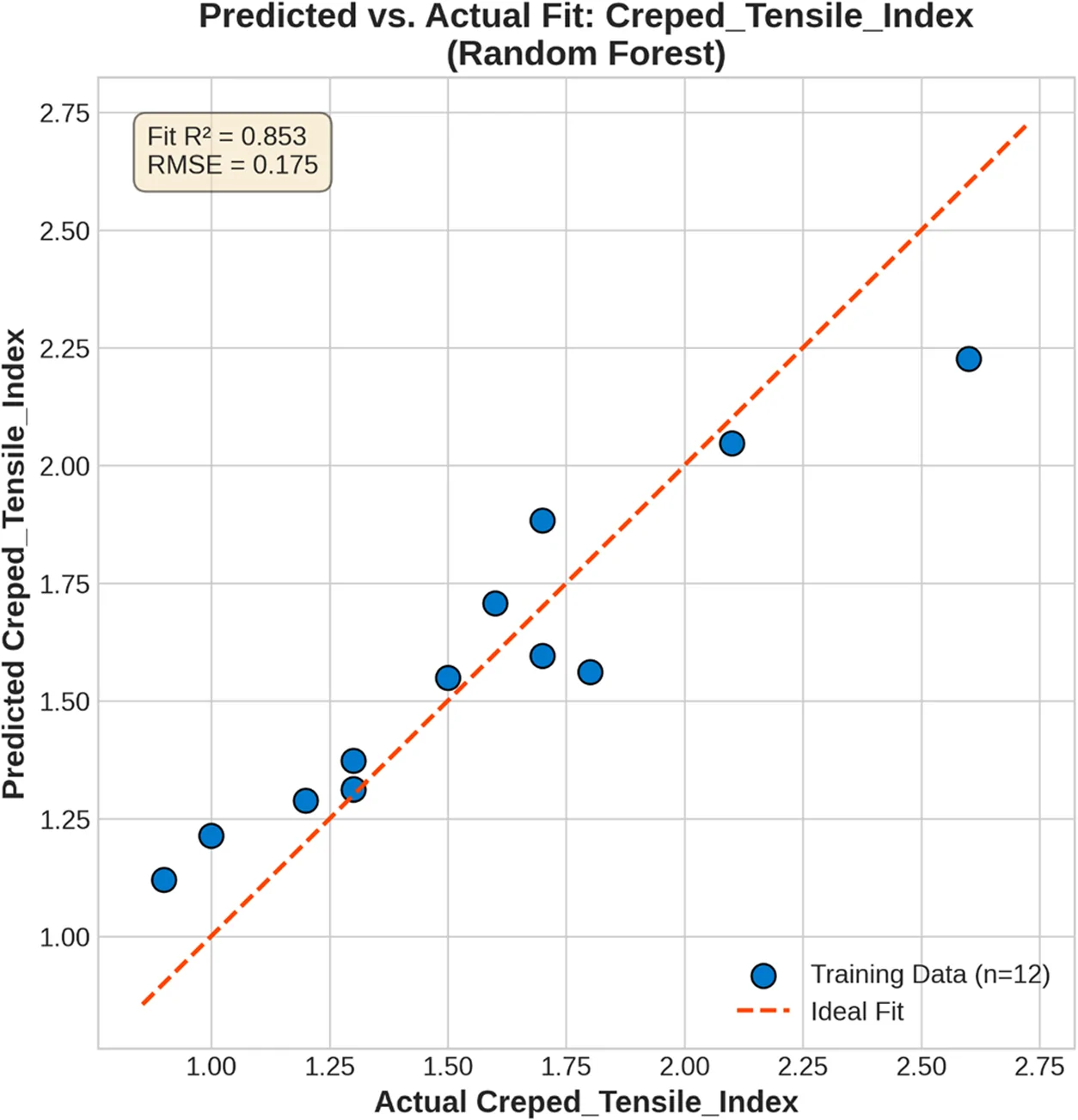
Intermediate model performance for creped tensile index prediction. Predicted versus measured creped tensile index (N·m/g) from the intermediate Random Forest regressor, using uncreped sheet properties and engineered feedstock features as inputs; shown as a representative example of the four intermediate property models.

The SHAP feature attribution analysis provided useful diagnostic insights into the primary variables associated with model outputs. For the intermediate models (Step 1), the uncreped properties were the dominant features associated with their respective creped counterparts. For instance, SHAP analysis identified uncreped Tensile_Index as the most influential feature in the prediction of Creped_Tensile_Index, a physically intuitive correlation that aligns with handsheet mechanics (Figure S7).

The predicted properties derived from the intermediate models were then used as variables to predict creped softness. The final softness model (Step 2), the SHAP analysis (summarized in the feature importance plot) revealed a more complex interplay of features. The most significant findings were (Figure 9).

**Figure 9:**
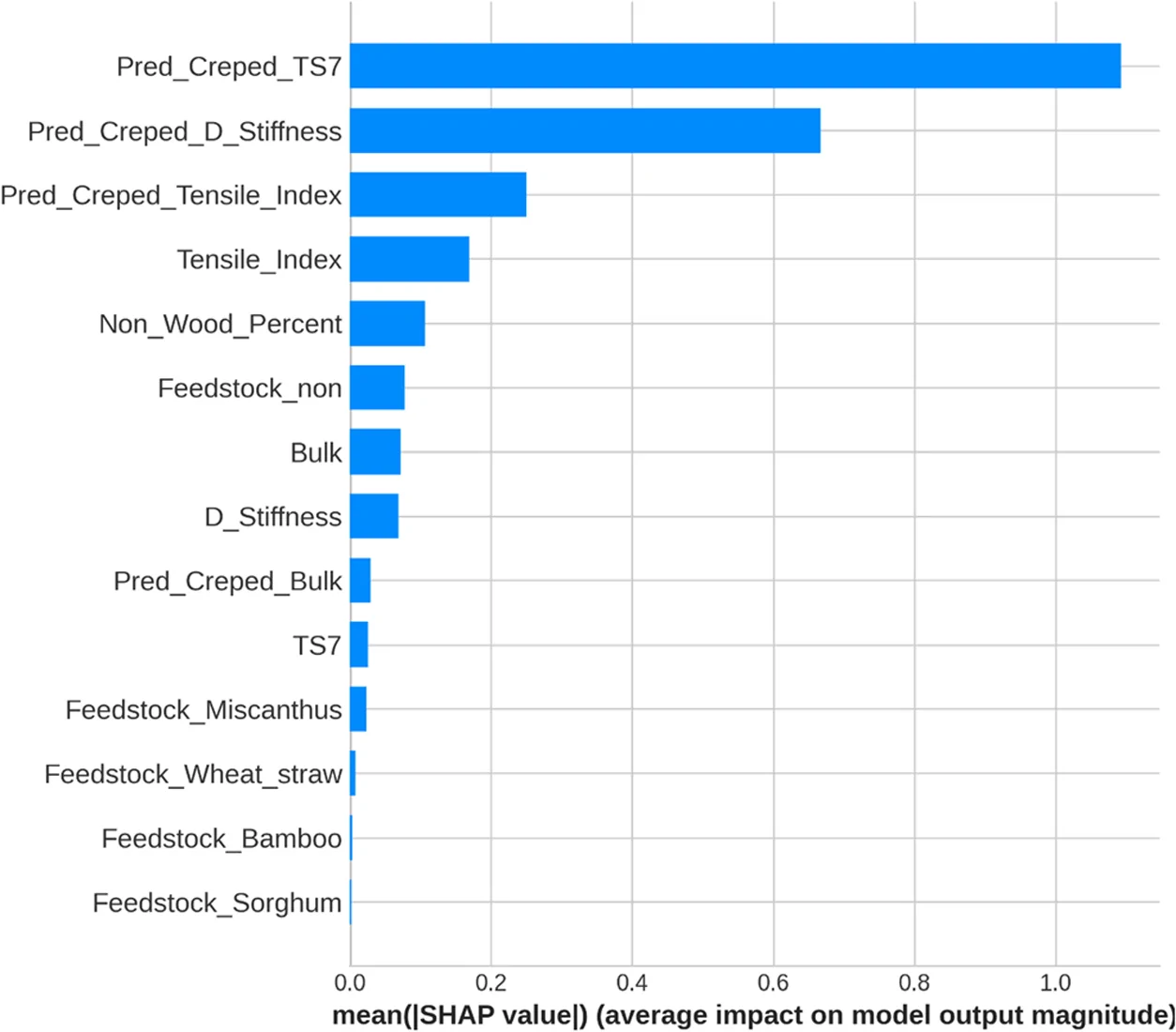
Feature importance for final softness prediction. SHapley Additive exPlanations (SHAP) analysis showing the mean absolute SHAP value for each input feature in the final chained Random Forest model predicting handfeel (HF, TPII).

The features most strongly associated with predicted HF_TPII were the intermediate creped properties generated in Stage 1, particularly Pred_Creped_D_Stiffness (Figure S11) and Pred_Creped_TS7 (Figure S8). This correlation aligns with the physical logic of the chained modeling structure, indicating that the model relies primarily on intermediate estimates of postcreping stiffness and fiber-level softness to predict final handfeel. Concurrently, feedstock variables also exhibited notable attribution weights. The binary indicator Feedstock_non, representing conventional wood-only furnishes, showed a positive association with predicted softness, whereas Feedstock_Wheat_straw was associated with a negative offset. However, because fiber type is highly collinear with fiber coarseness, curl, and sheet bulk, this correlation does not indicate that feedstock origin directly drives the softness outcome; rather, the model captures an associative pattern that reflects underlying morphological differences.

Even in the final model, some original uncreped properties, such as Bulk and TS7, retained measurable attribution values. This result indicates that while intermediate creped property estimates represent the dominant features in the model, precursor uncreped sheet descriptors provide complementary associative information for the final output.

#### 3.4.3 Challenges and Opportunities

This study’s primary limitation is its small sample size (*n* = 12), which restricts the statistical power of the regression models. However, for generalization, broader commercial furnish matrices and industrial creping conditions should be used. While Leave-One-Out Cross-Validation (LOOCV) was implemented to mitigate training bias, the limited sample size inherently bounds model stability. Additionally, SHAP analysis indicates that the model’s learned patterns correlate with established papermaking mechanisms; however, these attributions represent correlation rather than physical causation. The utility of this modeling approach is exploratory and hypothesis-generating. Future work must encompass substantially larger furnish libraries and replicate trials across broader nonwood substitution ranges before data-driven models can be considered for industrial formulation or quantitative prediction. (Salas et al., 2026; Zhou et al., 2025).

While LOOCV (Table S1) provides the most robust validation possible under these circumstances, the high cross-validation error standard deviation suggests the model’s reliability can vary across furnishes. The high in-sample *R* ^2^ fits, while encouraging, should be interpreted with caution, as they are likely inflated due to the model’s ability to fit a small number of points closely.

Nonetheless, the average RMSE (Figure S16) achieved through cross-validation suggests that the chained regression framework could be valid to represent the physical transitions in the tissue papermaking process. These exploratory proof-of-concept results underscore that data set expansion across broader blending matrices is a prerequisite before nonlinear machine learning architectures can provide demonstrable utility in industrial settings.

## 4 Conclusions

This study demonstrates that fiber morphology plays a crucial role in tissue performance during creping. Slender fibers such as bleached softwood Kraft and bamboo APMP produce stronger sheets, whereas coarser, deformed fibers tend to increase bulk. The creping process fundamentally changes the paper’s structure, boosting bulk by 80–150% but decreasing tensile strength by 80–89%. Blending 15–30% nonwood fibers with traditional wood pulps improves the balance among strength, absorbency, and softness. An exploratory machine learning framework was evaluated on this 12-furnish data set to examine relationships between precursor sheet properties and creped softness. Benchmarking demonstrated that the chained Random Forest model did not outperform a simple linear regression baseline. SHAP analysis highlighted the uncreped tensile index, fiber softness (TS7), and bulk as key features aligned with papermaking mechanisms. Overall, the empirical findings demonstrate that creping response is strongly associated with the mechanical and morphological state of the uncreped sheet, underscoring the importance of upstream fiber selection and furnish blending in engineering creped tissue structures.

## Supplementary Material



## Notes

Correlation matrix of uncreped and creped handsheet properties (Figure S1); predicted-versus-actual, residual, and SHAP analyses for creped bulk, tensile index, TS7, D stiffness, and handfeel (HF TPII) predictions (Figures S2–S15); the learning curve showing mean absolute error as a function of training sample size (Figure S16); leave-one-out cross-validation data for HF TPII prediction (Table S1); comparison of chained and single-stage machine-learning models (Table S2); and one-way ANOVA and Tukey HSD posthoc analyses of furnish effects on key tissue properties (Table S3a,b) (PDF)

## Abbreviations

APMP: alkaline peroxide mechanical pulping
BEK: bleached eucalyptus kraft
NBSK: northern bleached softwood kraft
WS: wheat straw
BB: bamboo
MS: miscanthus
SG: switchgrass
SH: sorghum
TSA: tissue softness analyzer
TS7: tissue softness parameter (fiber-level surface softness)
TS750: tissue softness parameter (surface smoothness)
HF: handfeel
TPII: total handfeel (softness) index
ML: machine learning
RF: random forest
SHAP: SHapley Additive exPlanations
LOOCV: leave-one-out cross-validation
RMSE: root mean squared error
PVA: poly­(vinyl alcohol)
XPS: X-ray photoelectron spectroscopy
CSF: canadian standard freeness
PFI: PFI mill refining unit
SOP: sorted office paper (recycled fiber grade)
LDC: light dry creping
