# Supplementary material for: Effect of Wood and Non-Wood Pulp Fiber Morphology on Laboratory-Simulated Tissue Paper Creping Performance: Functional Properties and Explainable AI
Source: ACS Omega. 2026 Oct 6;11(41):63918–32. doi: 10.1021/acsomega.6c08586 (PMC13688575; doi:10.1021/acsomega.6c08586)
Supplement: Supplementary file 1 [file ao6c08586_si_001.pdf]

Effect of Wood and Non-Wood Pulp Fiber Morphology on Laboratory-Simulated Tissue Paper Creping Performance: Functional Properties and Explainable AI

Fernando Urdaneta[1], Ronald Marquez[2*], Raine Viitala[3], Jorge Franco[1] , Isabel Urdaneta[1], Daniel Saloni[1], Joel J. Pawlak[1], Hasan Jameel[1], Ronalds W. Gonzalez[1*]

[1]Sustainable and Alternative Fibers Initiative, Department of Forest Biomaterials, North Carolina State University, Raleigh, NC, 27695-8005, USA.

[2]LEPAMAP-PRODIS Research Group, University of Girona, C/ Maria Aur` elia Capmany, 61, 17003, Girona, Spain

[3]Department of Mechanical Engineering, School of Engineering, Aalto University, P. O. Box 14400, Aalto, FI-00076, Finland

(*) Corresponding authors: R. Marquez (ronald.marquez@udg.edu): R. Gonzalez (rwgonzal@ncsu.edu).

Supplementary information

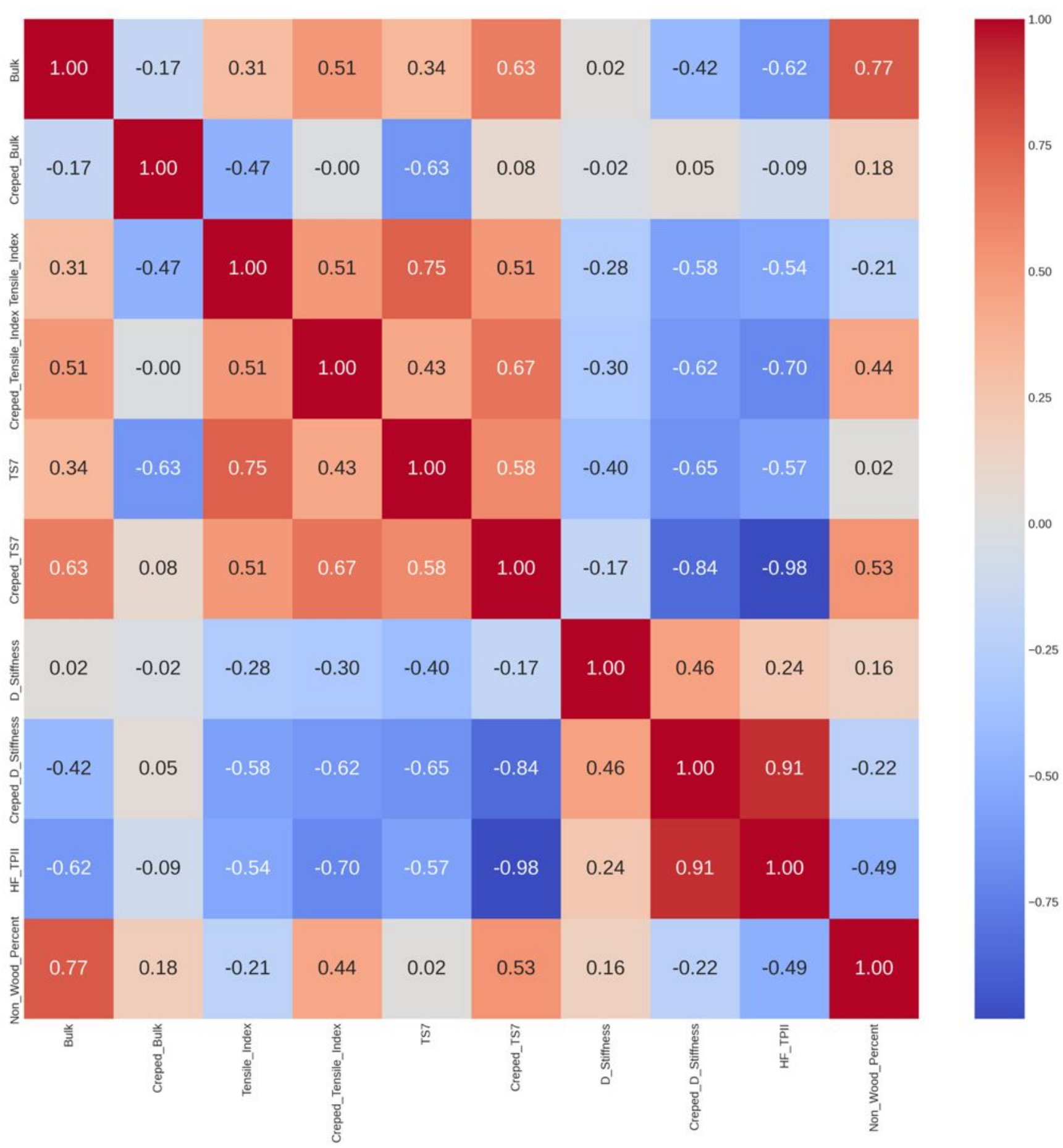

Figure S1 Correlation matrix of uncreped and creped handsheet properties. This matrix illustrates the statistical relationships between the physical properties of uncreped and creped handsheets, such as bulk, tensile index, and

stiffness. It supports the study's central hypothesis that uncreped sheet properties are strong predictors of how the paper will respond to the creping process

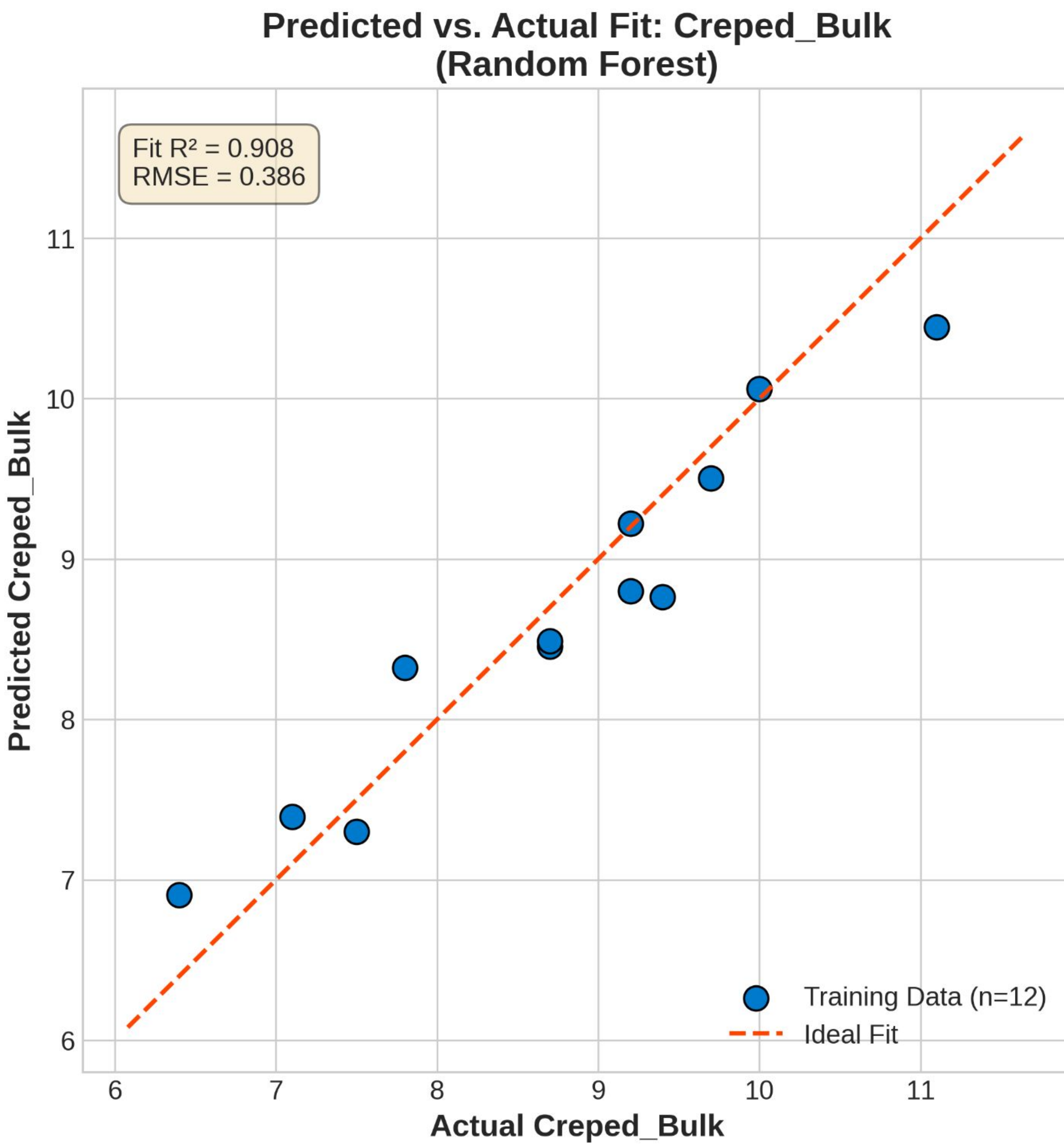

Figure S2 Predicted creped bulk vs actual creped bulk. A scatter plot demonstrating the performance of the Random Forest regression model in predicting the bulk of creped tissue sheets based on uncreped inputs. The alignment with the diagonal indicates the model's accuracy in capturing how different non-wood APMP fiber blends expand during creping.,

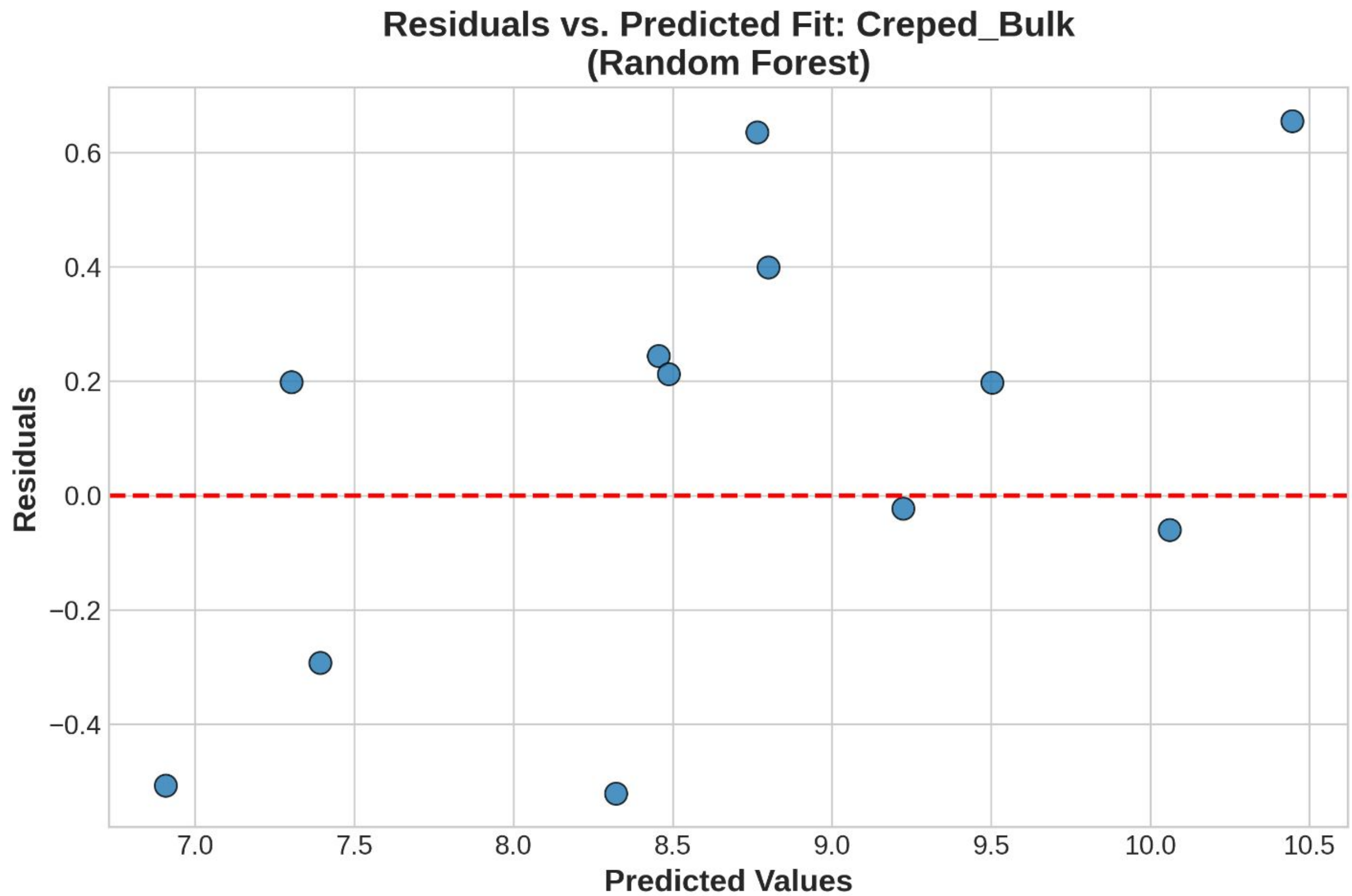

Figure S3 Residuals of creped bulk prediction. This plot shows the difference between the actual measured bulk and the values predicted by the machine learning model. It is used to validate the model's reliability and ensure there are no systematic biases across the range of experimental fiber substitutions

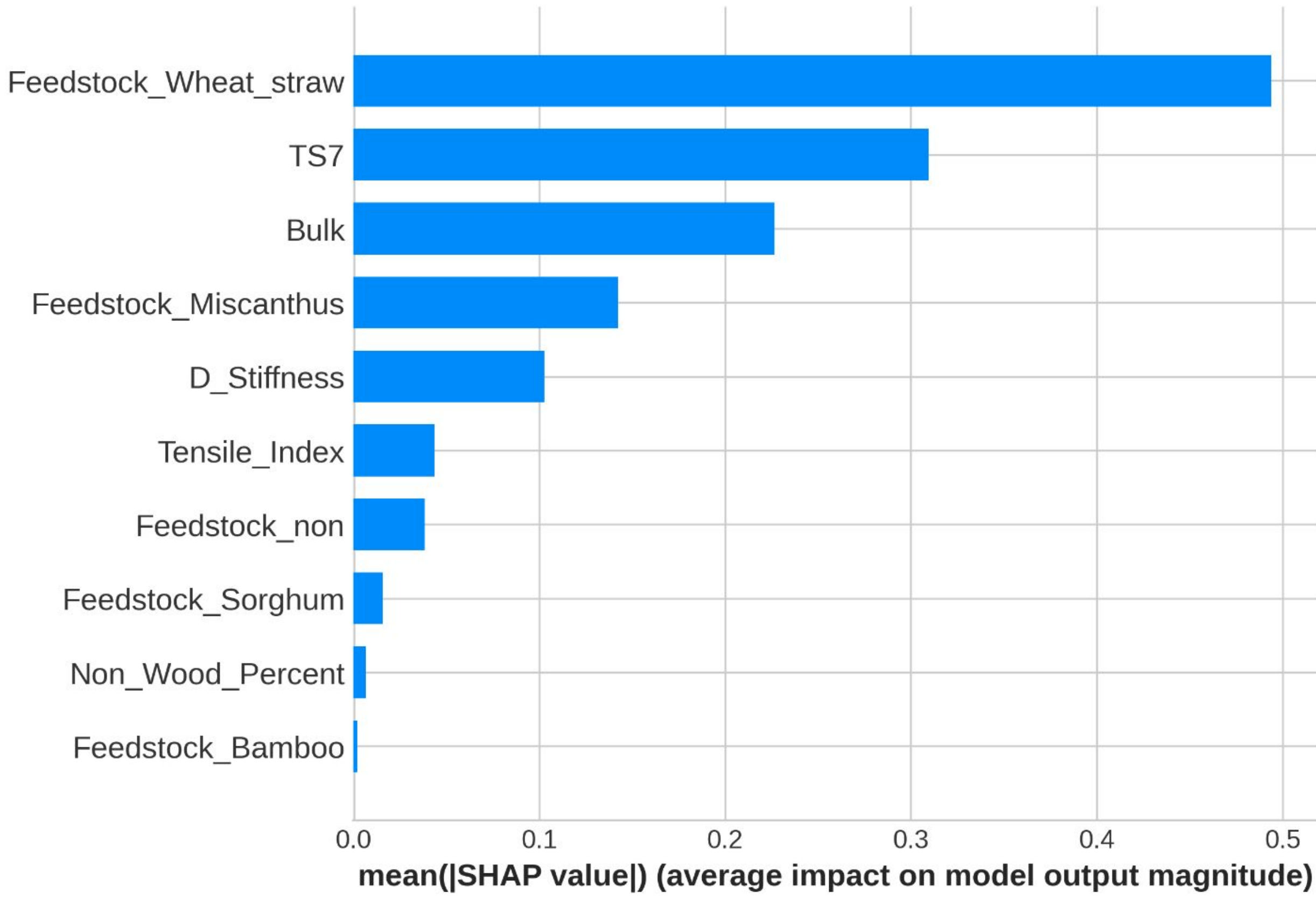

Figure S4 SHAP analysis of variables used to predict bulk. A SHapley Additive exPlanations (SHAP) chart identifying which features most significantly impacted the model's bulk predictions. This analysis highlights the relative importance of factors like uncreped bulk and fiber morphology (e.g., stiffness and coarseness) on the final creped structure

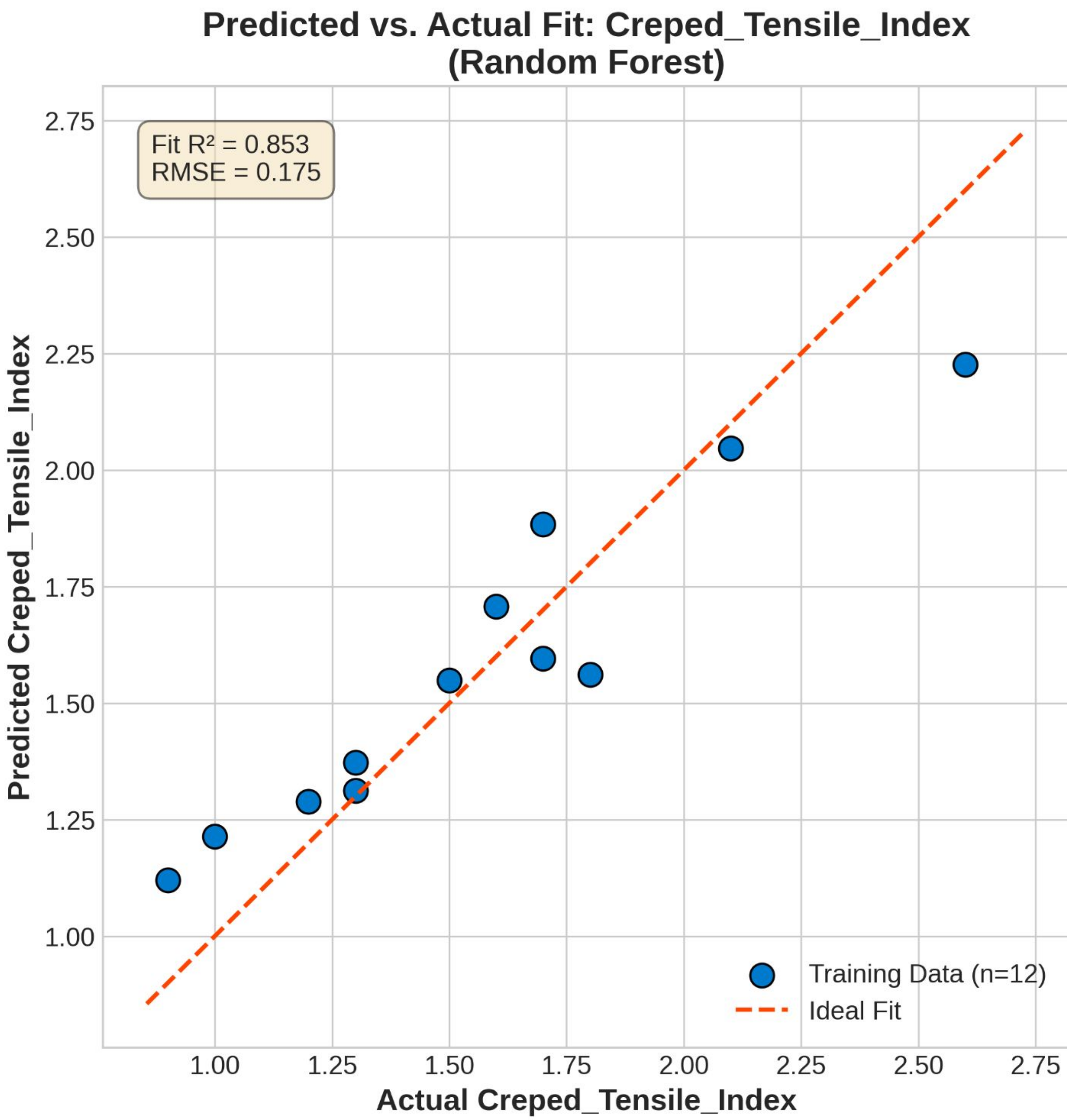

Figure S5 Predicted creped tensile index vs actual creped tensile index. This plot compares the machine learning model's predicted tensile strength against laboratory measurements. It evaluates the model's ability to forecast the 80-89% reduction in strength typically caused by the creping process across various non-wood blends

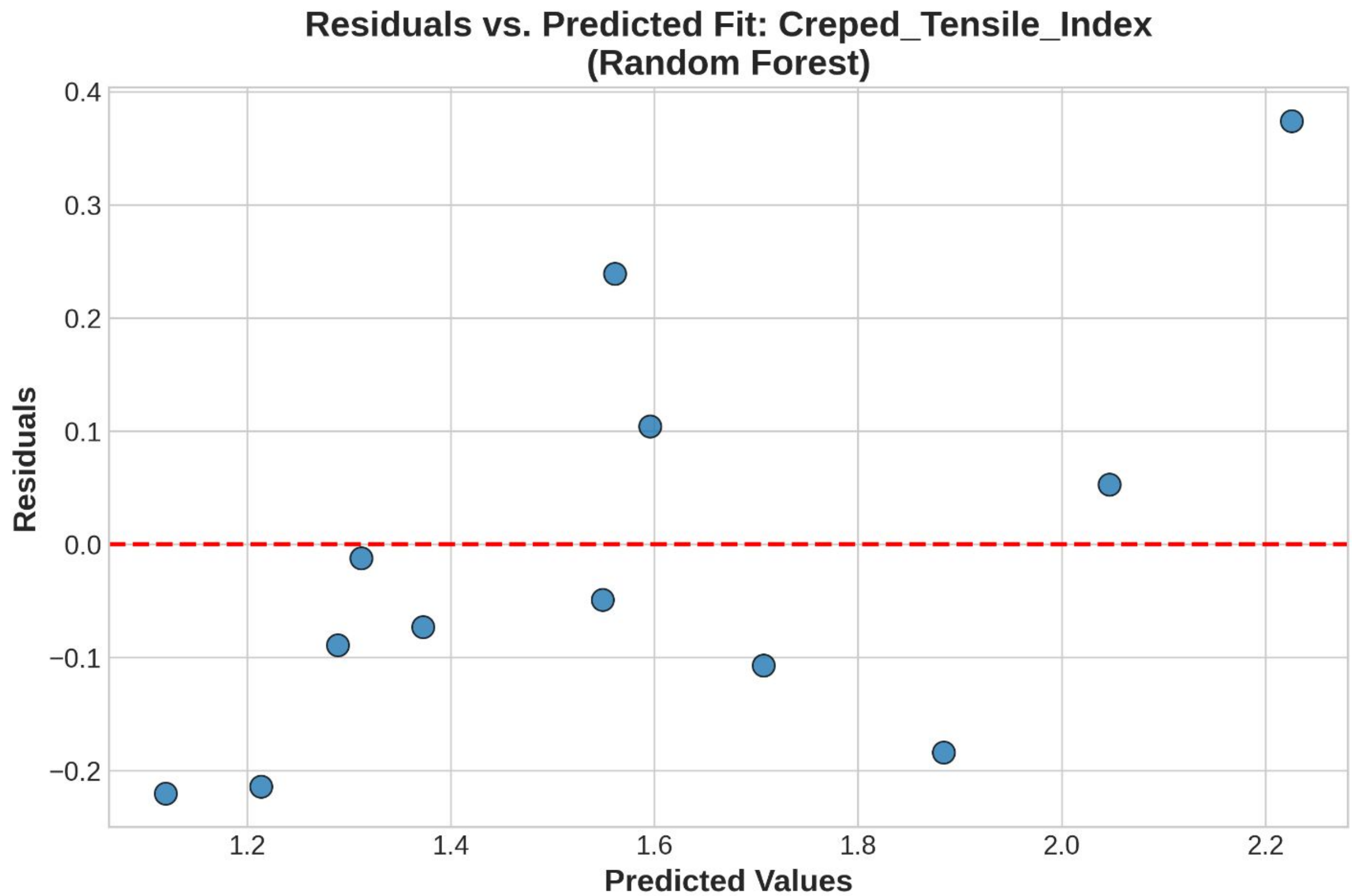

Figure S6 Predicted creped tensile index residuals. Residual analysis for the tensile index model, confirming the consistency of predictions for both high-strength (e.g., bamboo) and high-bulk (e.g., wheat straw) fiber furnishes

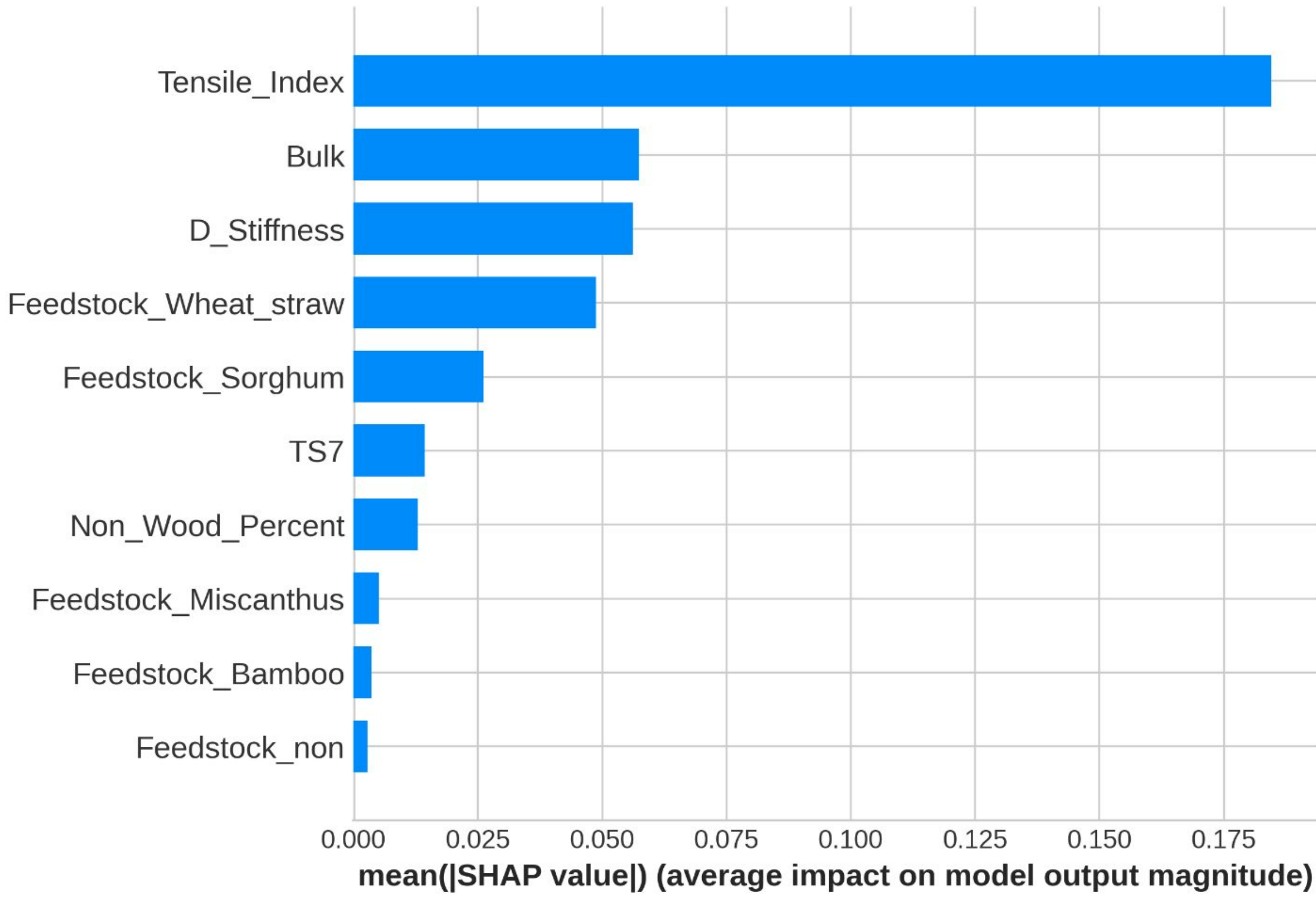

Figure S7 SHAP analysis of variables used to predict creped tensile index. This chart ranks the influence of input variables on the predicted strength of creped sheets, validating that uncreped tensile strength is a dominant predictor of resistance to buckling and fiber debonding

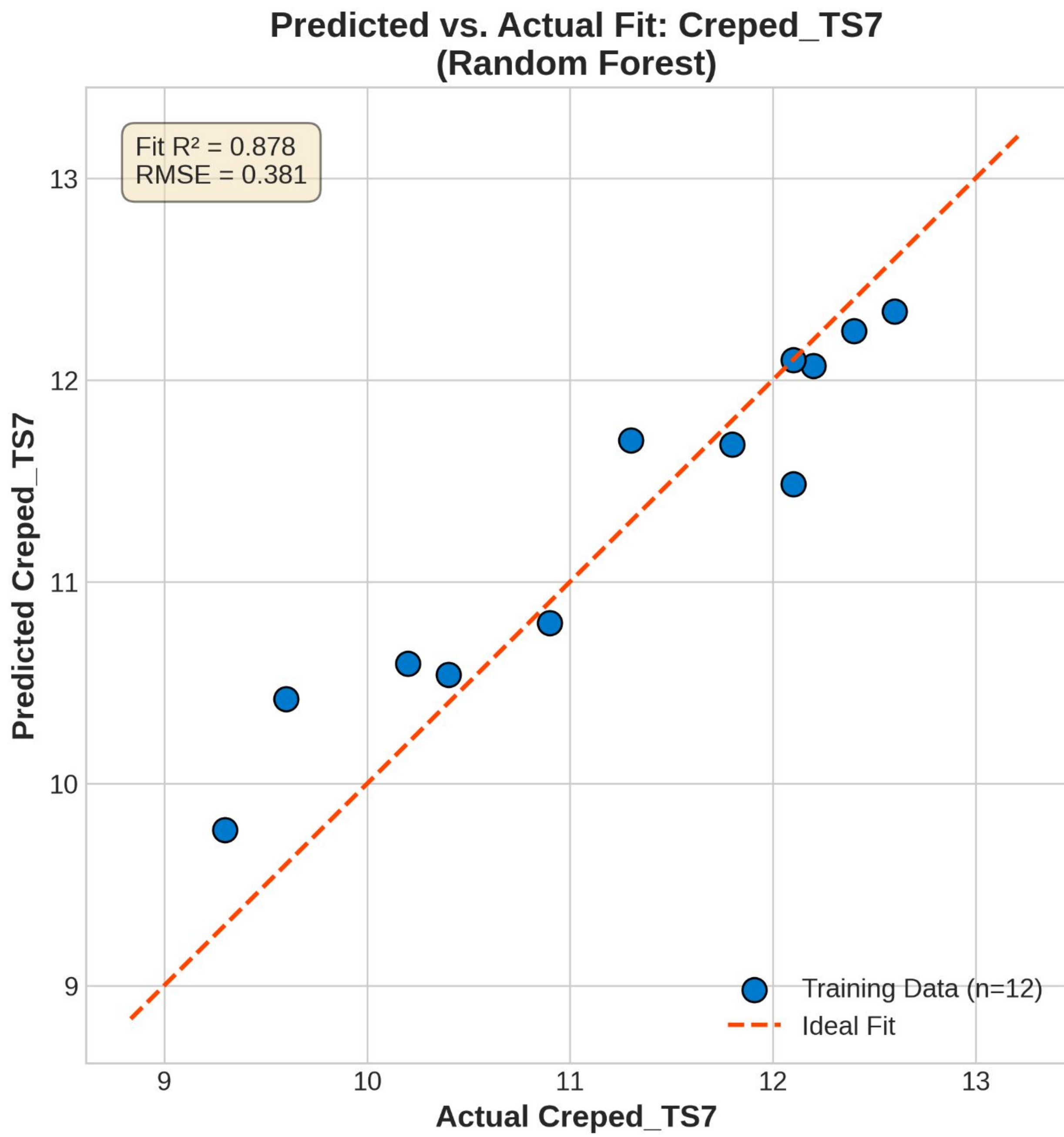

Figure S8 Predicted creped TS7 vs actual creped TS7.

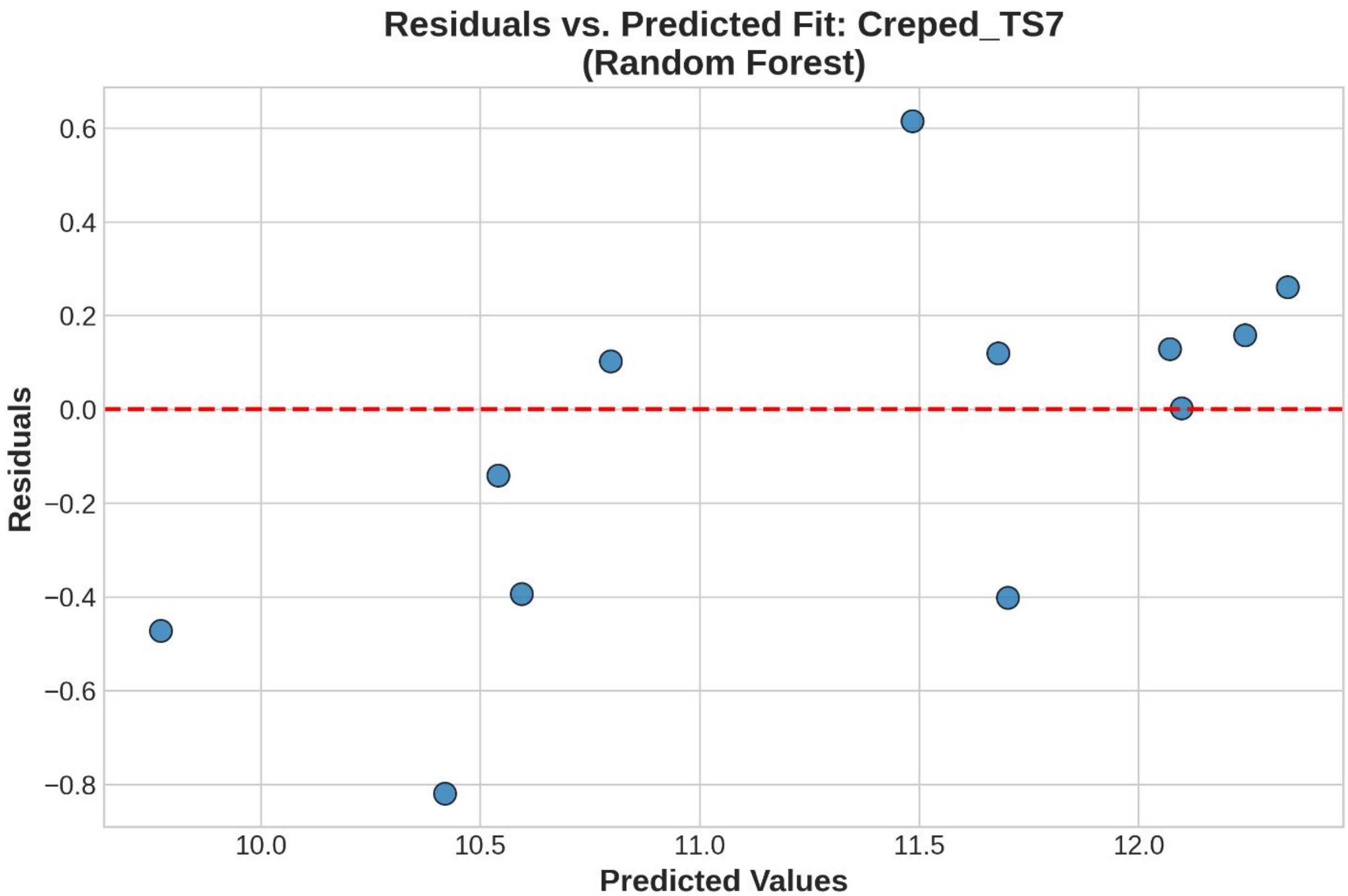

Figure S9 Predicted creped TS7 residuals. Residual plot for the TS7 softness metric, ensuring accurate modeling of the surface feel across furnishes with different fiber populations and fines contents

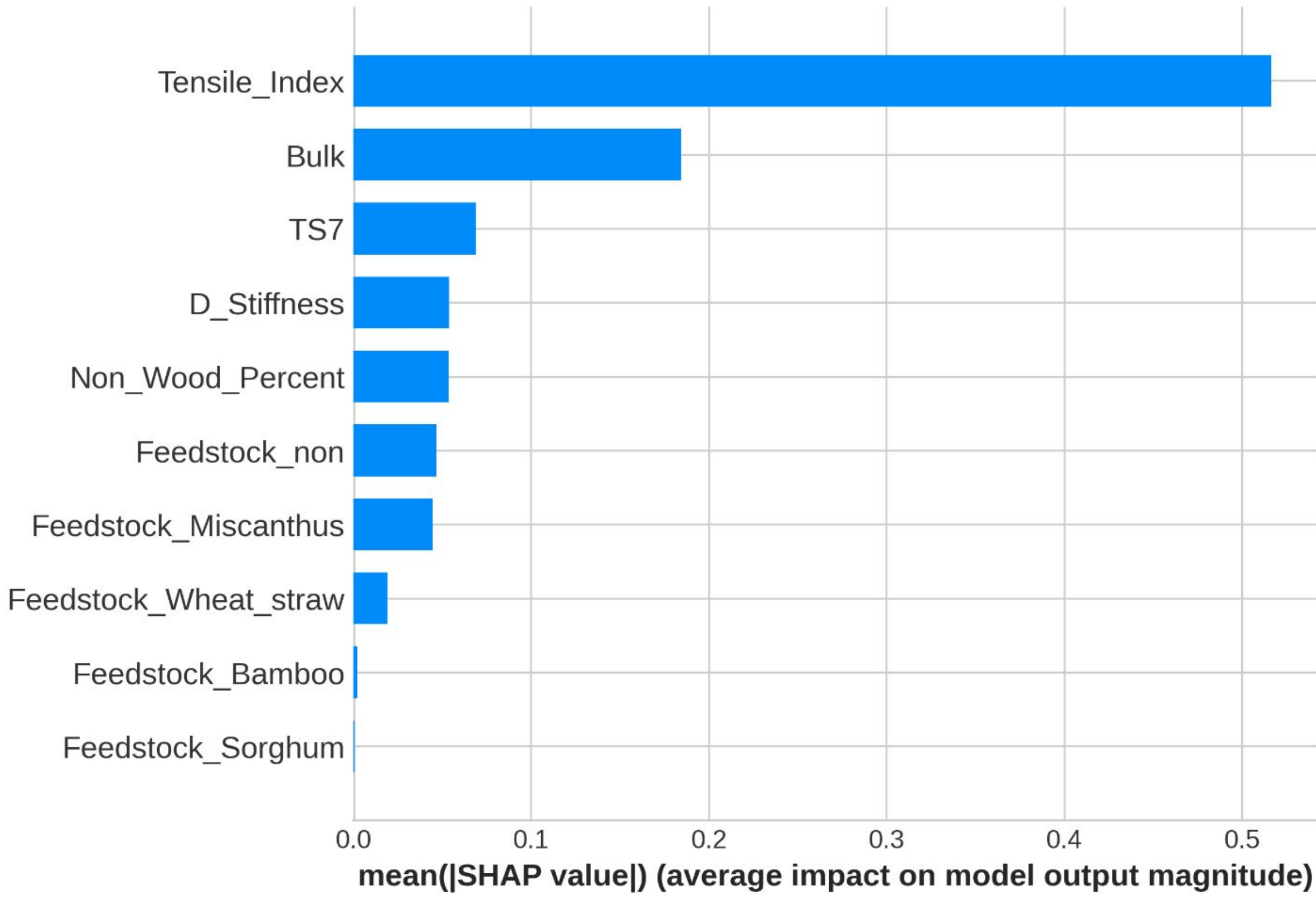

Figure S10 SHAP analysis of variables used to predict TS7. Analysis identifying the key drivers for fiber-level softness, emphasizing the role of fiber morphology and initial uncreped surface characteristics

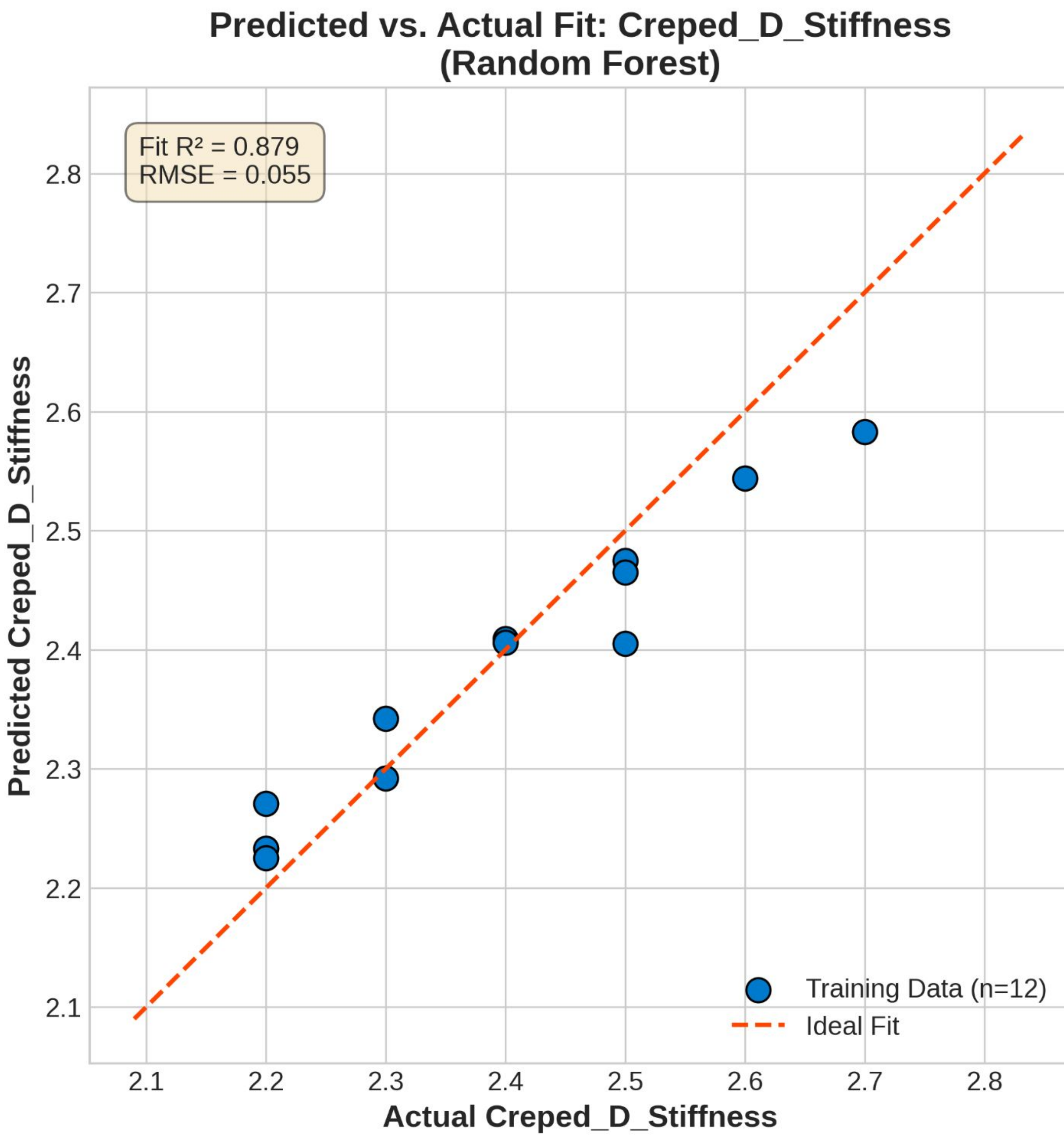

Figure S11 Predicted creped D "stiffness" vs actual creped D "stiffness". Evaluation of the model's capacity to predict the final structural stiffness (D) of the creped sheet, a critical component of the overall perceived handfeel

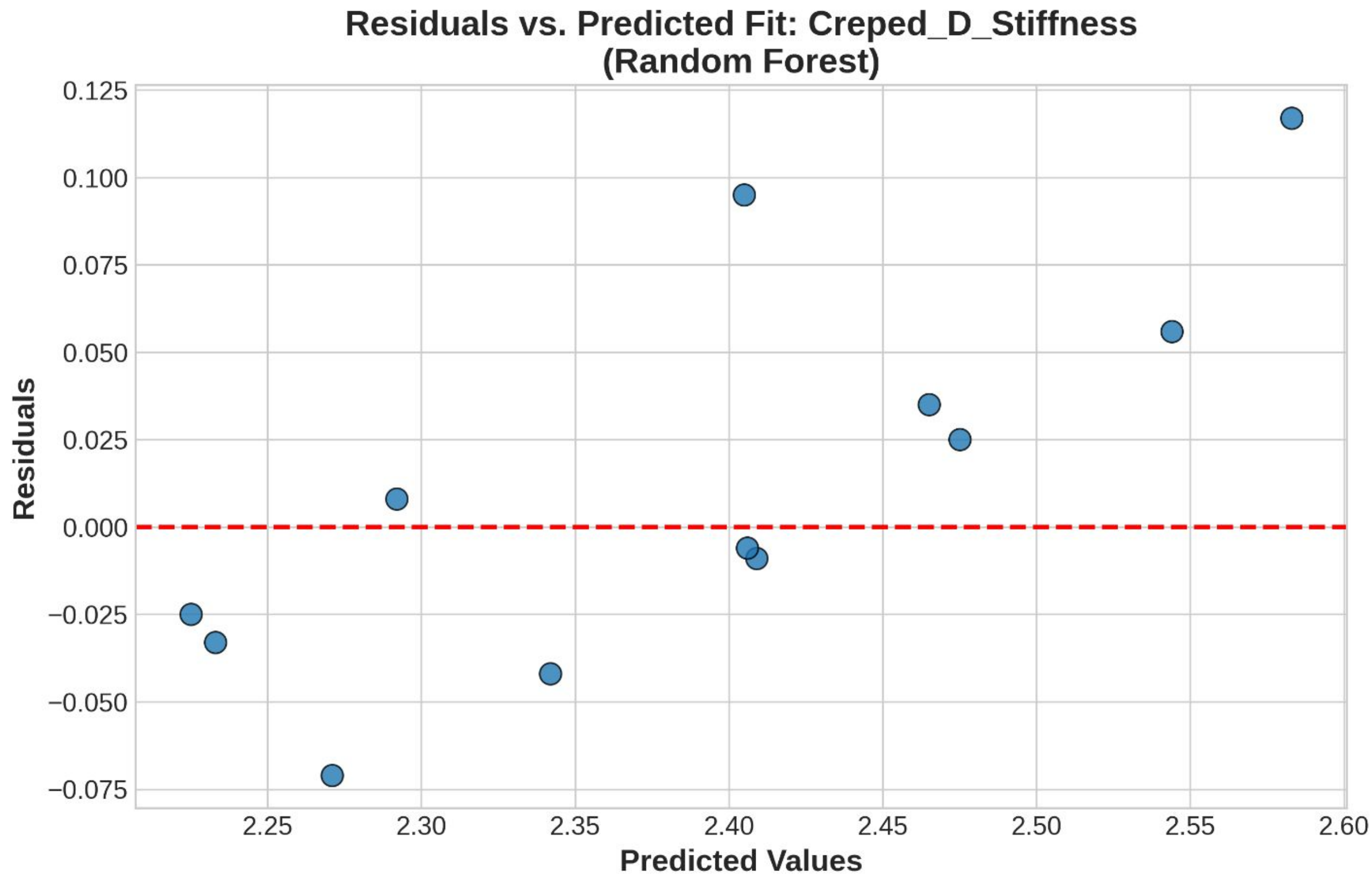

Figure S12 Predicted D residual values. This plot validates the lack of systematic error in the stiffness predictions, supporting the use of the model for diverse lignocellulosic feedstocks.

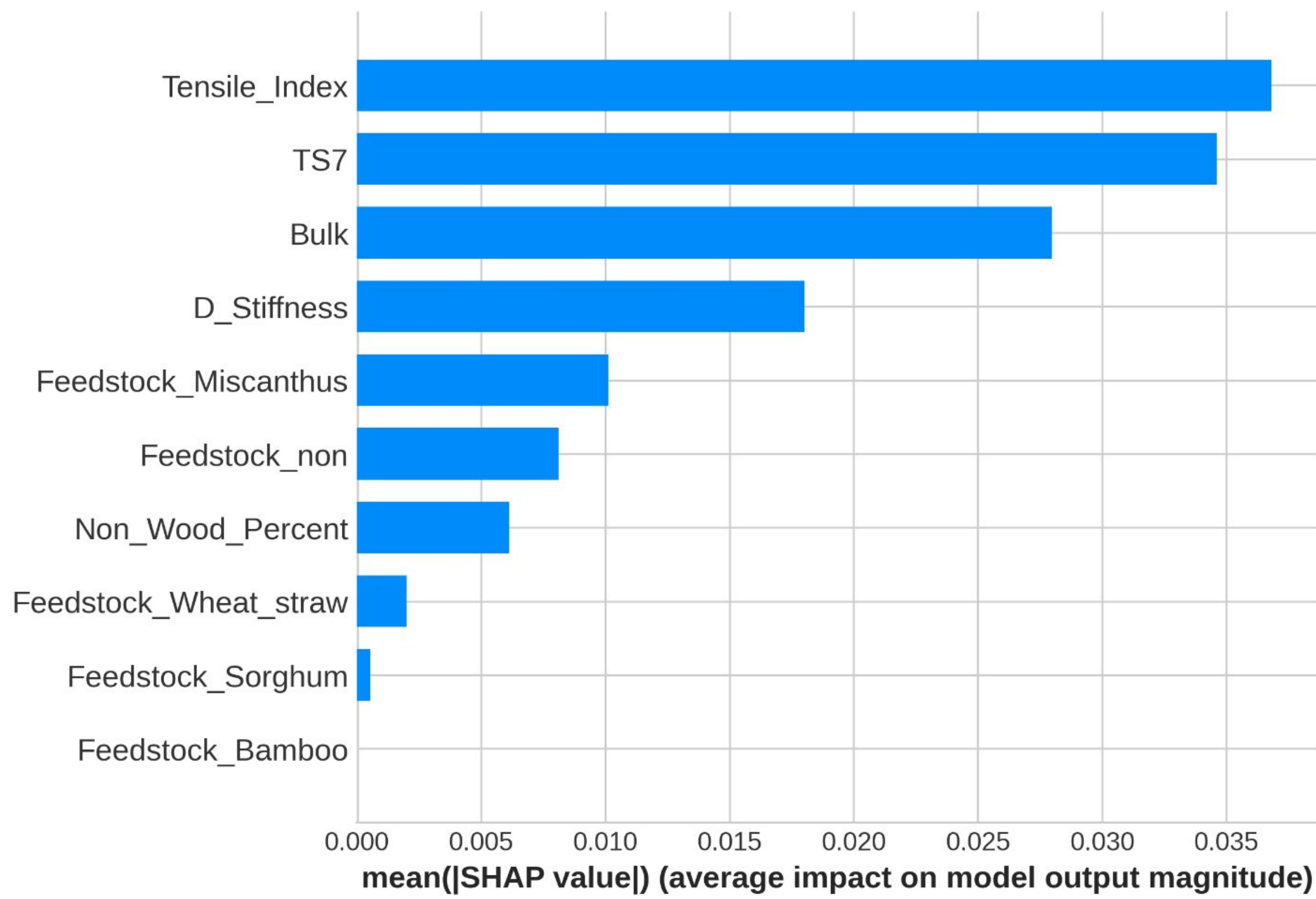

Figure S13 SHAP analysis of variables used to predict D. Highlights the variables that contribute most to the predicted stiffness of the tissue, such as fiber stiffness and the intensity of the creping response.

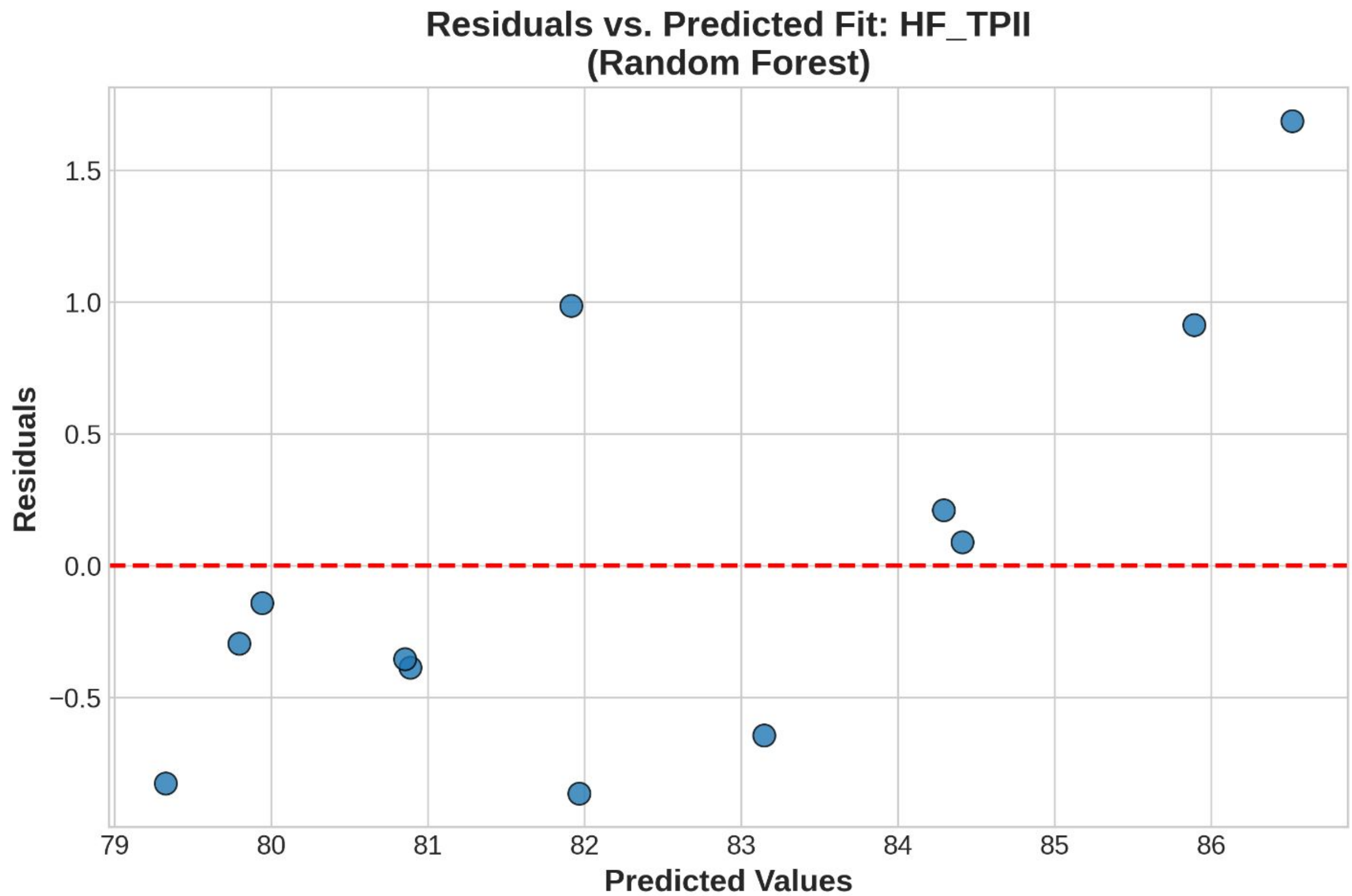

Figure S14 Residuals for predicted HF TPII. The error distribution for the final Handfeel (HF) Total Perceived Softness (TPII) predictions, demonstrating the reliability of the chained machine learning approach.

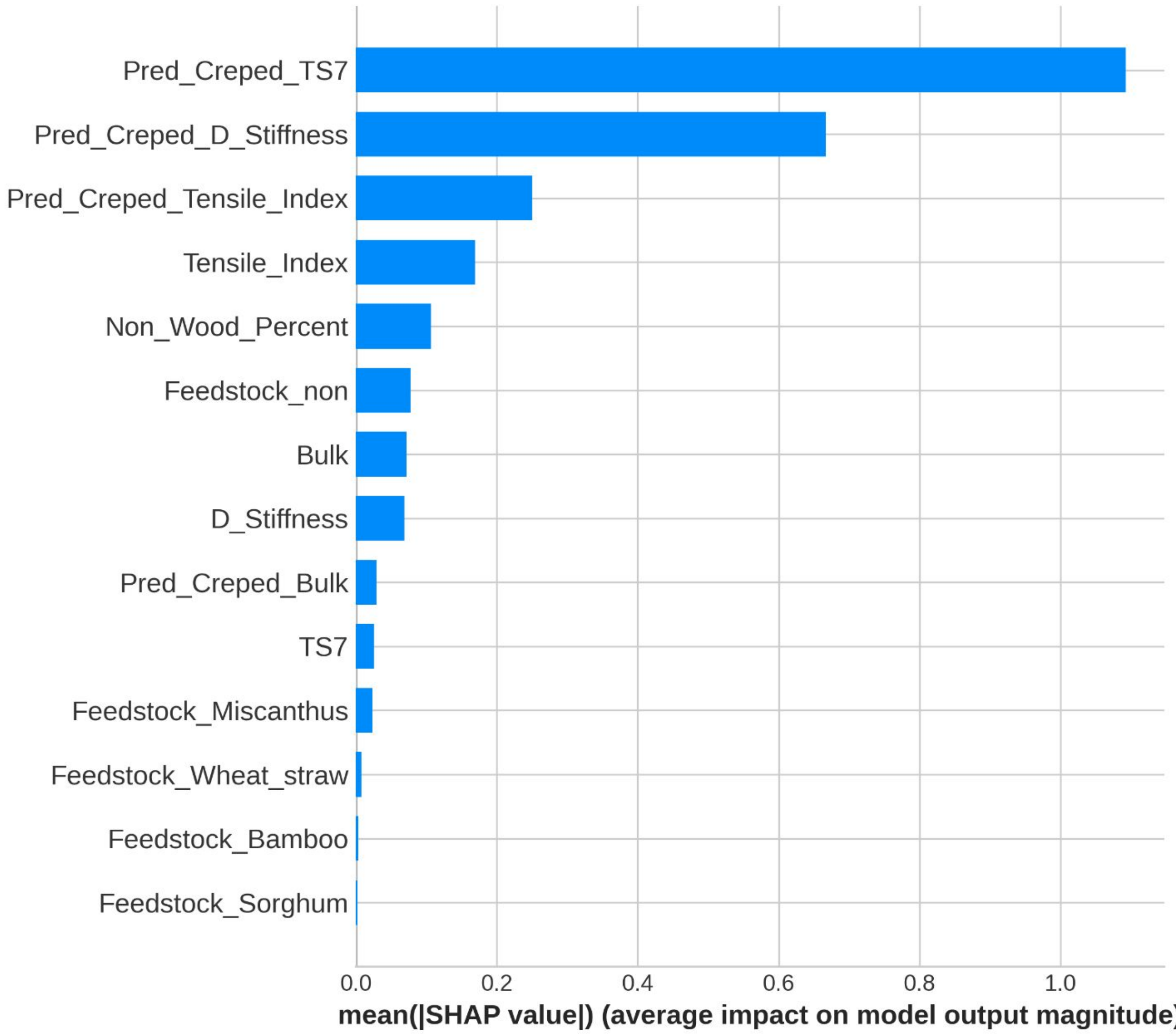

Figure S15 SHAP analysis for variables used to predict HF TPII. This summary chart reveals the most influential features for the final softness prediction (TP2), showing that uncreped tensile index, TS7, and uncreped bulk are the primary drivers of quality in the final bioproduct

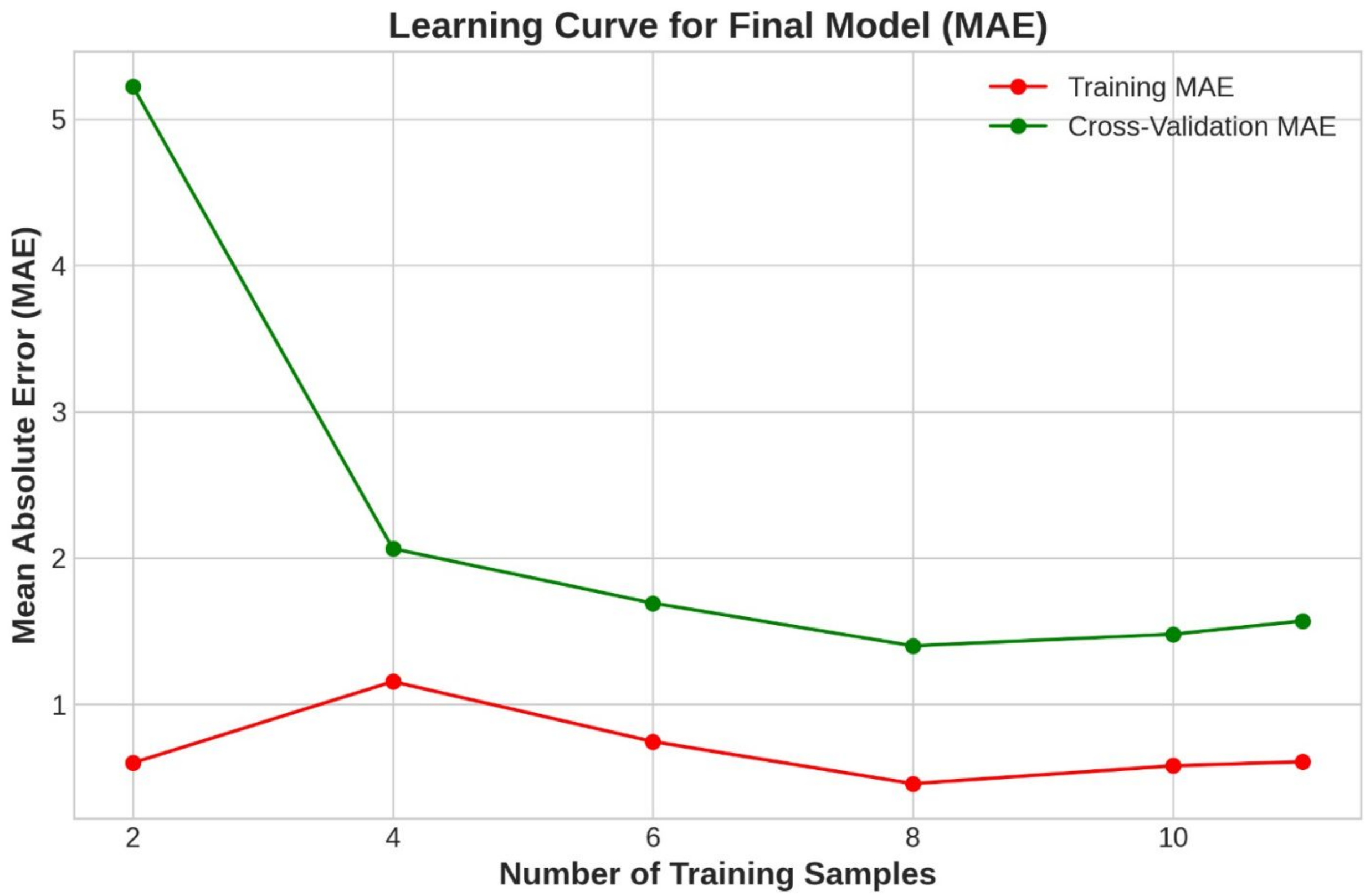

Figure S16 Mean absolute error (MAE) vs number of training samples. A learning curve showing how the model's error decreases as more experimental data is included. This demonstrates the potential of ML to reduce the need for extensive physical prototyping in R&D cycles

Table S1 Leave one out report data. aw validation data for the handfeel (HF TPII) prediction model. It lists actual versus predicted values and the individual error for each sample, providing a transparent view of the model's accuracy during cross-validation.

F_TPII	Predicted_HF_TPII	Error
88.20	84.70	3.50
86.80	84.45	2.35
79.50	80.74	-1.24
78.50	80.65	-2.15
80.50	81.39	-0.89
82.50	83.34	-0.84
84.50	84.24	0.26
81.10	83.30	-2.20
84.50	82.98	1.52

79.80	80.07	-0.27
82.90	80.11	2.79
80.50	81.34	-0.84

Table S2 Model Comparison

Model	LOOCV MAE
Chained Random Forest (final model)	1.570
Single-stage Random Forest (uncreped → HF_TPII)	2.182
Single-stage Linear Regression (uncreped → HF_TPII)	1.451

One-way ANOVA and Tukey HSD post-hoc analysis of furnish effects on key tissue properties

Statistical comparisons were performed on the mean and standard deviation values reported in Table 3 for all 24 furnishes. Tests assume n = 5 independent handsheets per furnish, consistent with standard practice for this measurement protocol (Urdaneta et al., 2024); exact replicate counts for this specific dataset were not separately recorded.

Table S3a. Overall ANOVA results

Property	F-statistic	df (between, within)	p-value	Tukey HSD critical difference
Tensile index (N·m/g)	189.31	23, 96	< 0.001	0.256
Bulk (cm³/g)	44.92	23, 96	< 0.001	0.956
HF_TPII (softness)	5.83	23, 96	< 0.001	14.688

Table S3b. Selected Tukey HSD pairwise comparisons discussed in the main text

Table S3b. Selected Tukey HSD pairwise comparisons discussed in the main text

Comparison	Property	Mean A	Mean B	Difference	p (adjusted)	Significant?

BEK55-NBSK15-SH30 vs. 100BEK	HF_TPII	58.7	88.2	29.5	< 0.001	Yes
BEK55-NBSK15-SH30 vs. BEK85-NBSK15	HF_TPII	58.7	86.8	28.1	< 0.001	Yes
BEK55-NBSK15-SH30 vs. BEK85-BB15	HF_TPII	58.7	89.2	30.5	< 0.001	Yes
BEK70-NBSK15-SH15 vs. 100BEK	HF_TPII	72.7	88.2	15.5	0.027	Yes
BEK85-BB15 vs. BEK70-NBSK15-BB15	HF_TPII	89.2	79.8	9.4	> 0.05	**No**
BEK85-BB15 vs. BEK55-NBSK15-BB30	HF_TPII	89.2	79.0	10.2	> 0.05	**No**
